# Novel Phosphotidylinositol 4,5-Bisphosphate Binding Sites on Focal Adhesion Kinase

**DOI:** 10.1371/journal.pone.0132833

**Published:** 2015-07-17

**Authors:** Jun Feng, Blake Mertz

**Affiliations:** The C. Eugene Bennett Department of Chemistry, West Virginia University, Morgantown, West Virginia, United States of America; Oregon State University, UNITED STATES

## Abstract

Focal adhesion kinase (FAK) is a protein tyrosine kinase that is ubiquitously expressed, recruited to focal adhesions, and engages in a variety of cellular signaling pathways. Diverse cellular responses, such as cell migration, proliferation, and survival, are regulated by FAK. Prior to activation, FAK adopts an autoinhibited conformation in which the FERM domain binds the kinase domain, blocking access to the activation loop and substrate binding site. Activation of FAK occurs through conformational change, and acidic phospholipids such as phosphatidylinositol 4,5-bisphosphate (PIP_2_) are known to facilitate this process. PIP_2_ binding alters the autoinhibited conformation of the FERM and kinase domains and subsequently exposes the activation loop to phosphorylation. However, the detailed molecular mechanism of PIP_2_ binding and its role in FAK activation remain unclear. In this study, we conducted coarse-grained molecular dynamics simulations to investigate the binding of FAK to PIP_2_. Our simulations identified novel areas of basic residues in the kinase domain of FAK that potentially undergo transient binding to PIP_2_ through electrostatic attractions. Our investigation provides a molecular picture of PIP_2_-initiated FAK activation and introduces promising new pathways for future studies of FAK regulation.

## Introduction

Focal adhesions (FA) are integrin-mediated protein complexes that are found peripheral to the cell membrane at sites of cell attachment to the extracellular matrix (ECM) [1,2]. Focal adhesions establish a direct mechanical link between the actin cytoskeleton and the ECM and also act as cellular signaling integrators, sensing biochemical and mechanical stimuli from the extracellular environment. Diverse and critical biological events, including cell migration, proliferation, differentiation, and survival, are mediated by proteins localized to focal adhesions [3].

**Fig 1:**
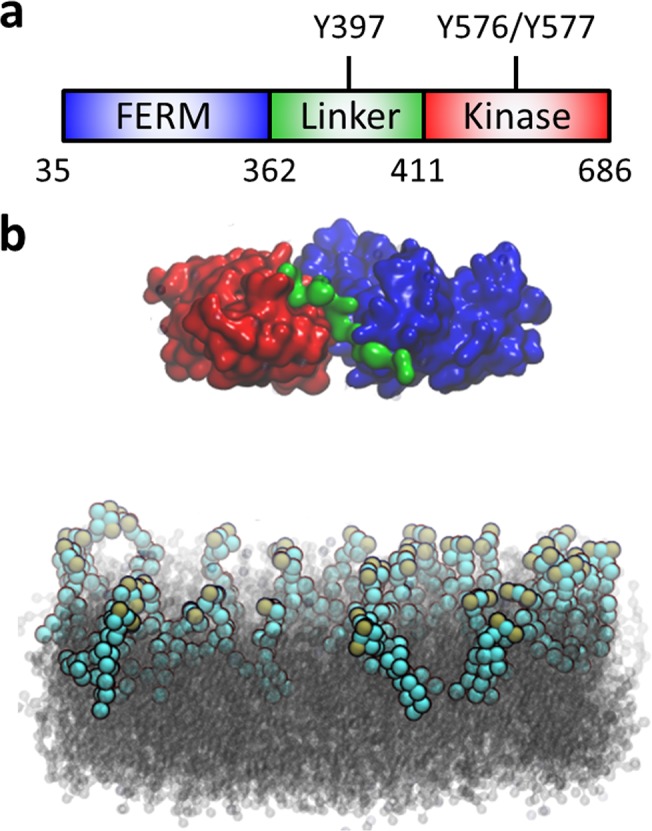
Domain structure of FAK and illustration of the simulation system. (a) The two N-terminal domains of FAK form the autoinhibited conformation and are connected via a linker. FERM domain: *blue*; kinase domain: *red*; linker: *green*. (b) FAK lies above a DOPC bilayer (*gray*) with 10% PIP_2_ (*spheres*, cyan and ochre) in the upper leaflet. Ions and water molecules are omitted for clarity. All molecular graphics are rendered in VMD [13].

Focal adhesion kinase (FAK) is an essential component in the macromolecular assembly of focal adhesions, as well as a crucial kinase that participates in a variety of cellular signaling pathways [4]. FAK is involved in several developmental processes including angiogenesis [5] and axonal outgrowth [6]. In addition, dysregulation of FAK activity is linked to numerous pathological events such as tumorigenesis and metastasis [7]. Understanding the structural underpinnings of FAK activation is essential to development of therapeutic treatments for these diseases. FAK is composed of 4 major domains: an N-terminal band 4.1, Ezrin, Radixin, Moesin (FERM) domain, a central tyrosine kinase domain followed by an unstructured proline-rich region, and a C-terminal focal adhesion targeting (FAT) domain. The FERM and kinase domains are separated by a linker containing the autophosphorylation site, Y397 (**Fig 1**). The FERM domain plays an essential autoinhibitory role in the regulation of the catalytic activity of FAK [8], in which the F2 subdomain contacts the C-lobe of the kinase domain, blocking access to the catalytic cleft and the activation loop (A-loop) [9]. As a result, the kinase domain remains locked in an inactive, unphosphorylated conformation. In addition, the linker between the FERM and kinase domains forms a beta strand that is part of a beta sheet within the F1 lobe, preventing autophosphorylation at Y397. It has been proposed [9,10] that a conformational change in the autoinhibited form of the FERM-kinase domain is the key initial step in FAK activation, exposing Y397 within the linker to phosphorylation [11] and subsequent recruitment of Src. Once Src binds to the linker, it phosphorylates two tyrosines within the exposed A-loop (Y576 and Y577) and renders FAK fully active [12].

FERM-interacting molecules, including growth factor receptors, p53, Ezrin, and acidic phospholipids, are known to play key roles in FAK regulation [14]. In particular, phosphatidylinositol 4,5-bisphosphate (PIP_2_) directly binds FAK in the FERM domain, inducing a conformational change and subsequent activation [10,15]. While PIP_2_ binding is likely driven by electrostatic forces [16], the molecular details of FAK binding to PIP_2_, the stoichiometry of binding, and the binding kinetics remain unknown. Determination of these binding characteristics is essential in order to understand PIP_2_-mediated activation of FAK.

We performed a series of modeling studies to investigate the molecular details of interactions between FAK and PIP_2_. Coarse-grained molecular dynamics (CGMD) [17,18] allows modeling of length and timescales that are significantly larger than all-atom molecular dynamics and are ideal for phenomena such as PIP_2_ activation of FAK. CGMD simulations were carried out to examine the binding of FAK to bilayers containing PIP_2_ lipids. Binding to the KAKTLRK basic patch within the FERM domain was observed, in agreement with experimental studies [10,15]; surprisingly, we also identified PIP_2_ binding in the kinase domain. These results provide novel insight into the mechanisms controlling FAK activation, and could potentially identify new strategies to manipulate FAK regulation and treat disease.

## Computational Methods

FAK simulations were performed using the crystal structure of the autoinhibited conformation [9] (PDB 2J0J). Missing residues in the A-loop of the kinase domain (residues 574–583) were modeled as a random coil and further minimized using the molecular simulation package CHARMM c37b1 [19]. Missing residues from the linker region (residues 363–393) were not modeled due to its length and structural flexibility. The all-atom model was converted to a coarse-grained model with the MARTINI v2.2 force field [20], and backbone C_α_ atoms were constrained by an elastic network [21] with a cutoff of 0.9 nm and a force constant of 500 kJ-mol^-1^. Simulation of FAK in water was initially carried out with the FERM and kinase domains allowed to move independently of one another. Deformation of the global structure occurred (results not shown), limiting all subsequent simulations with the FERM and kinase domains constrained as a single unit in the autoinihibited form. Another 1-μs-long simulation of FAK in water with the added constraint was carried out to verify that the system would remain stable.

To model the PIP_2_-containing lipid bilayer, we used an equilibrated DOPC bilayer of 15.2 nm × 15.2 nm in the x-y dimension and randomly replaced 10% of the DOPC molecules on the upper leaflet of the bilayer with PIP_2_, as per experimental studies [15], resulting in a lipid bilayer system composed of 629 DOPC molecules and 33 PIP_2_ molecules. Finally, FAK was placed at distances of 4.5 nm (simulation I), 2.5 nm (simulation II), and 1.5 nm (simulation III), respectively, from the surface of the lipid bilayer, and 170 sodium ions and ≈27,000 polarized water molecules were added to the simulation box to make the final dimension of each system 15.2 nm × 15.2 nm × 19.1 nm with ≈92,000 particles (**Fig 1**).

All CGMD simulations were run in GROMACS v4.6 [22]. Simulations were performed in the isothermal-isobaric ensemble (*NPT*) with nonbond cutoff at 1.2 nm. Temperature was coupled at 320 K using a Berendsen thermostat with a coupling constant of 1 ps. The pressure was coupled to a Berendsen barostat at 1 bar with a relaxation time of 1 ps. Each simulation was run for 1 μs with an integration time step of 10 fs and system coordinates were collected every 10 ps. Given the relatively fast system equilibration and initial ligand-binding timescales, as well as the simulation length, the full trajectory is used for analysis unless stated otherwise. (Discussion of simulation convergence can be found in S5 Table.) Analysis was carried out with GROMACS analysis tools and custom-made scripts.

## Results

### A. FAK binds to PIP_2_-containing bilayers

**Fig 2:**
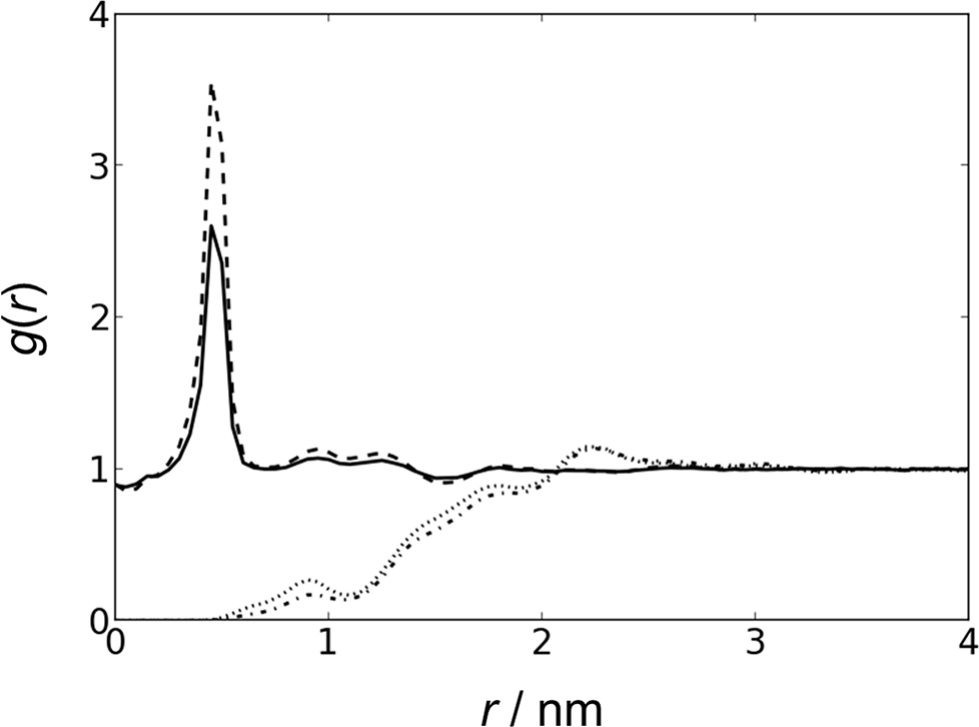
FAK does not actively recruit PIP_2_. RDF of ions as a function of distance to PIP_2_ in the presence (*solid line*) and absence (*dashed line*) of FAK. RDF of PIP_2_ as a function of distance to PIP_2_ in the presence (*dash-dotted line*) and absence (*dotted line*) of FAK. All distances are measured with respect to the 1’-phosphate of PIP_2_. The final 900 ns of each simulation was used for analysis. Simulations II and III had very similar results and are therefore not shown.

As a system validation, we first simulated FAK in polarized water. The protein was stable with an average RMSD of ≈0.3 nm. Our next set of simulations allowed FAK to freely diffuse above a lipid bilayer. Radial distribution functions (RDF) of PIP_2_ and ions to PIP_2_ with and without FAK were calculated (**Fig 2**), and there is no notable difference in distribution in the presence or absence of FAK. This result indicates that the presence of FAK does not globally perturb the distribution of PIP_2_ within the lipid bilayer, ruling out the possibility that FAK actively recruits large clusters of PIP_2_ lipids. However, it is possible for PIP_2_ to form clusters in the presence of divalent cations such as calcium, meaning that FAK could potentially interact with multiple PIP_2_ molecules [23].

**Fig 3:**
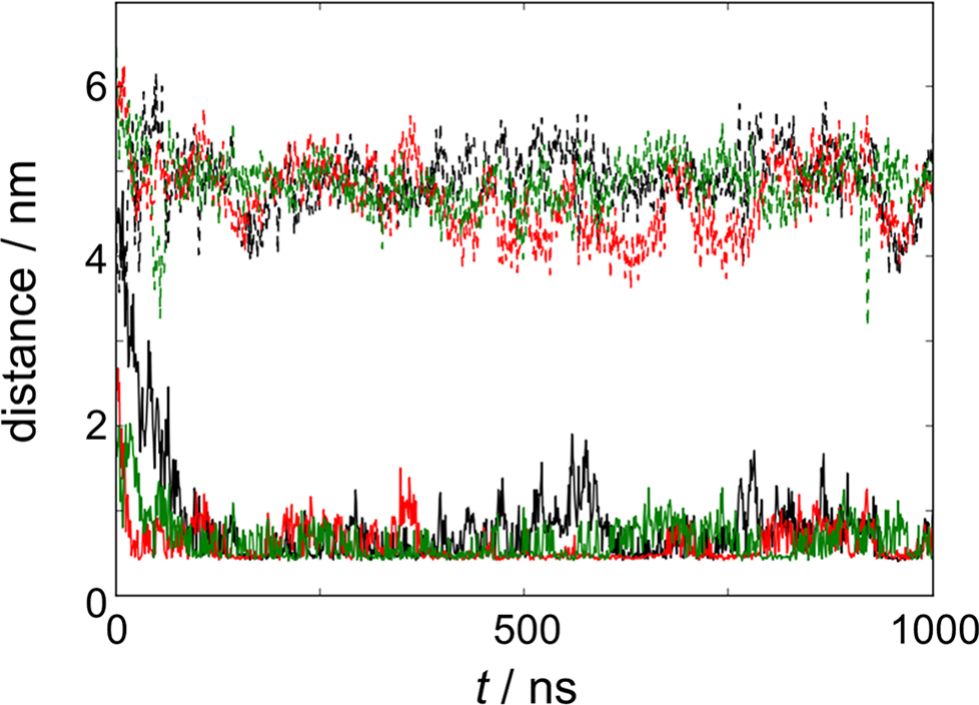
FAK preferentially interacts with the PIP_2_-containing bilayer leaflet. Minimal distance between FAK and DOPC leaflet with (*solid line*) and without PIP_2_ (*dashed line*) in simulation I (*black*), II (red), and III (*green*)

For all simulations, FAK was placed far enough from the lipid bilayer to ensure that binding to PIP_2_ would occur through random diffusion, independent of initial orientation. Due to the use of periodic boundary conditions, FAK could freely diffuse and interact with each leaflet of the lipid bilayer (one leaflet containing PIP_2_ and DOPC, the other leaflet containing only DOPC). This design was intentional, since PIP_2_ is asymmetrically distributed *in vivo* [24–26]. Our simulations showed that FAK preferentially interacted with the PIP_2_-containing leaflet (**Fig 3**).

### B. Multiple association and dissociation events occur between FAK and PIP_2_

We next examined the propensity for FAK to interact with PIP_2_ lipids (**Table 1**). FAK was considered to bind with PIP_2_ if the minimal distance between any coarse-grained bead of FAK and any phosphate bead of PIP_2_ was less than 0.52 nm. (Results are not sensitive to the cutoff—see S1–S4 Tables.) The average distance between PIP_2_ and FAK was 0.48 nm ±0.02 nm. FAK was bound to PIP_2_ for at least 2/3 of the total simulation time (**Table 2**), with initial binding events occurring at 80, 18, and 30 ns, respectively (**Fig 3**). Binding was dynamic, as multiple events of PIP_2_ association and dissociation were observed throughout the course of the simulations. In addition, when FAK was bound to PIP_2_, it can interact with multiple PIP_2_ lipids (usually between 1–3 molecules), as determined by the percentage of time when FAK binds *n*_P_ number of PIP_2_ lipids (*n*_P_ = 1–6) (**Table 2**).

**Table 1:** Comparison of PIP_2_ and PC lipids involved in FAK binding.

Simulation	% time of binding		*N*_*L*_ ^a^ (*x*_i_ > 10%)^b^		*N*_*L*_ (*x*_i_ > 5%)	
	PIP_2_	PC	PIP_2_	PC	PIP_2_	PC
I	68.1	31.8	9	0	12	7
II	88.2	58.4	8	1	13	17
III	84.6	39.1	7	0	12	1

^a^ *N*_*L*_ is defined as the number of lipids that make contact over 10% or 5% of simulation time.

^b^ The contact percentage of PIP_2_/PC lipids is calculated as the number of times a specific PIP_2_/PC lipid made contact with FAK normalized by the total number of times FAK established contact with PIP_2_/PC lipids.

**Table 2:** Quantification of FAK binding to PIP_2_.

Simulation	% time of binding^a^	*n*_P_ PIP_2_ bound to FAK, % time of binding^b^			
		1	2	3	4–6
I	68.1	40.5	35.5	17.6	6.4
II	88.2	28.7	41.0	24.1	6.3
III	84.6	38.9	37.3	17.9	5.9

^a^ Percentage of time FAK is bound to PIP_2_ during simulation.

^b^ Percentage of time FAK is bound to *n*_P_ number of PIP_2_.

### C. Observed PIP_2_ binding sites on FAK

To identify PIP_2_ binding sites on FAK, the percentage of time *x*_*i*_ each residue interacts with PIP_2_ is monitored using *x*_*i*_ = *n*_*i*_/*N*, where *n*_*i*_ is the number of times residue *i* makes contact with PIP_2_ and *N* is the total number of times FAK makes contact with PIP_2_ during the simulation (**Table 3**). All identified interacting residues are basic (lysine or arginine) and thus capable of establishing electrostatic attractions with the highly negatively-charged PIP_2_ head groups. Among these residues, we identified known PIP_2_-binding residues K216, K218, R221, and K222 (part of the KAKTLRK ridge) [15,27]. These residues lie on the surface of the F2 subdomain of the FERM domain and are critical for FAK activation. Surprisingly, we also observed additional basic residues not previously known to interact with PIP_2_, as will be detailed below.

**Table 3:** Percentage of time (*x*_*i*_) individual residues contact PIP_2._

Simulation	K191^a^	K216	K218	R221	K222	R229	R508	R514	K515	K578	K621	K627	R640	K657	R665
I	7.8	8.6	18.2	1.3	20.6	17.4	7.3	4.8	19.0	27.7	22.3	35.6	18.9	5.4	10.4
II	4.1	5.9	12.2	1.9	7.3	4.8	27.8	11.2	13.1	37.8	53.1	39.4	16.5	6.9	11.0
III	11.0	7.3	23.4	10.5	21.7	16.6	0.0	3.5	22.6	35.4	15.7	48.0	19.5	6.4	10.5

^a^ Residues with *x*_*i*_ > 5% in at least two simulations or *x*_*i*_ > 10% in any simulation.

**Fig 4:**
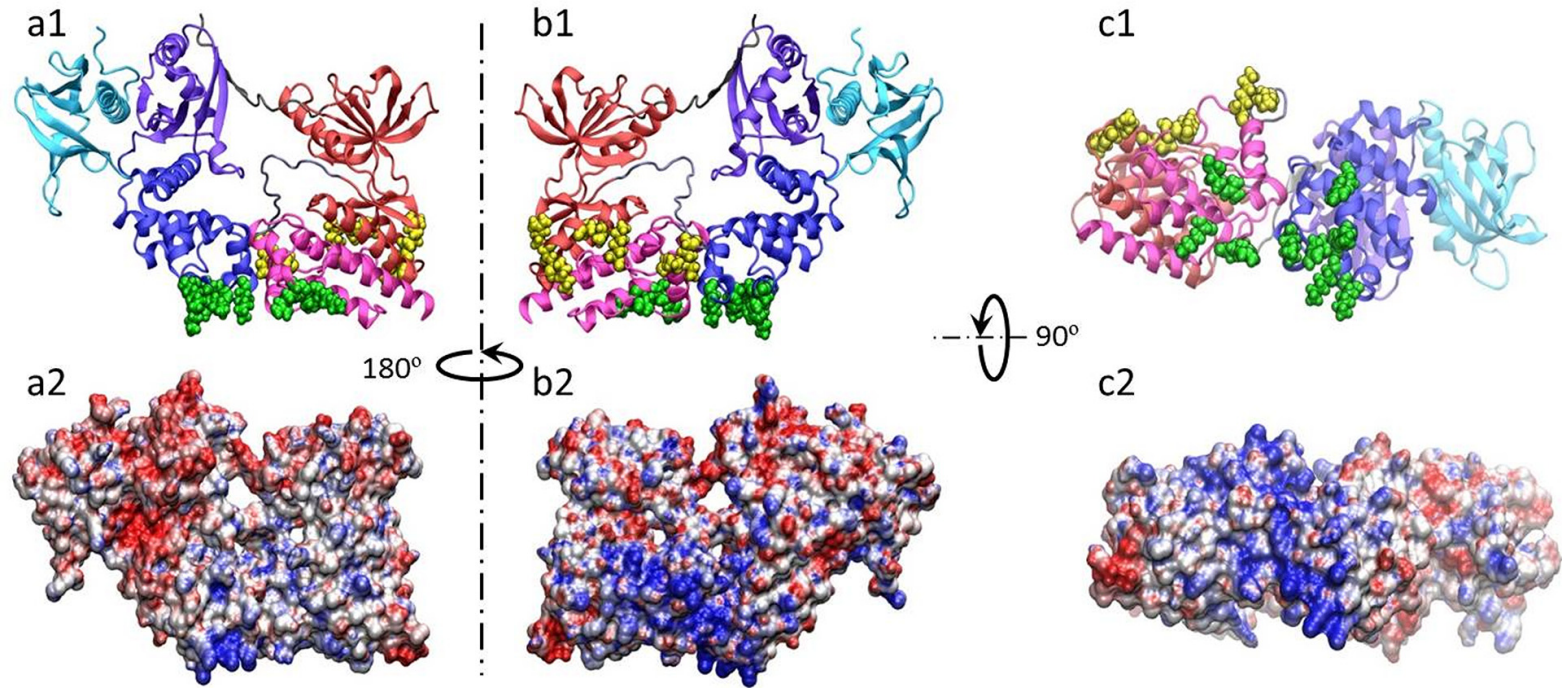
Group I and II interaction sites correspond to large basic patches in FAK. a1, b1, and c1: Mapping of Group I (*green*) and Group II (*yellow*) residues onto the crystal structure of FAK (F1 (*violet*), F2 (*blue*), and F3 (*cyan*) subdomains of the FERM domain, N-lobe (red), A-loop (*iceblue*), and C-lobe (*magenta*) of the kinase domain, and linker (*gray*)). a2, b2, and c2: Electrostatic potential surface of FAK kinase calculated using APBS [28]. The view of the electrostatic potential surface in a2, b2, and c2 corresponds to the orientation shown in a1, b1, and c1, respectively.

To further characterize the binding sites, PIP_2_-interacting residues were grouped based upon their location on the protein surface, designated Group I and Group II (**Table 4**and **Fig 4**). Group I residues are located at the interface between the F2 subdomain of the FERM domain along the functionally important KAKTLRK ridge [15,27] and directly adjacent in the C-lobe of the kinase domain. These two clusters in Group I (the KAKTLRK ridge and the C-lobe of the kinase domain) are proximal to each other and may form part of a larger phospholipid binding site. Group II residues are completely new and represent a potential binding site suggested by our simulations, located near the interface between the N- and C-lobes of the kinase domain. Both Group I and Group II binding sites are tightly correlated to the two basic patches on the electrostatic potential surface of the protein (**Fig 4**).

**Table 4:** Grouping of PIP_2_ interaction sites.

Group I	F2^a^	K191	K216	K218	R221	K222	R229
	C-lobe	R640	K657	R665			
Group II	N-lobe & C-lobe	R508	R514	K515	K578	K621	K627

^a^ F2 is part of the FERM domain, N- and C-lobes are part of the kinase domain.

### D. FAK adopts multiple binding poses with PIP_2_

**Fig 5:**
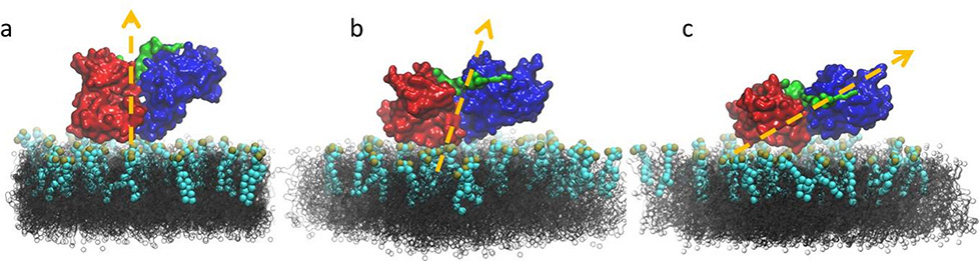
FAK adopts a set of preferential orientations toward PIP_2_-containing lipid bilayers. Arrows represent the direction from the center of mass of the F2 subdomain of the FERM domain and C-lobe of the kinase domain to the COM of the F1 subdomain of the FERM domain and N-lobe of the kinase domain. Color scheme is the same as Fig 1.

Group I and Group II interactions with PIP_2_ account for the majority of FAK-PIP_2_ contacts and are independent of one another (**Table 5**). However, preferential orientations of FAK with respect to the lipid bilayer do occur. Interactions involving only Group I required both the KAKTLRK ridge and C-lobe of the kinase domain to be effaced with the lipid bilayer (**Fig 5A**). When both Group I and Group II sites interacted with PIP_2_, a tilted orientation with the Group II site on the C-lobe facing the bilayer surface occurred (**Fig 5B**). Finally, when only Group II preferentially interacted with PIP_2_, it required an orientation in which the C-lobe was heavily tilted towards and almost parallel to the bilayer (**Fig 5C**)_._

**Table 5:** Percentage of time Group I and Group II sites contacted PIP_2_ during FAK-PIP_2_ interactions.

Simulation	Only Group I	Only Group II	I and II	Neither I or II
I	31.4	42.6	23.2	2.8
II	22.7	60.3	15.6	1.4
III	25.3	40.7	32.6	1.4

## Discussion

The computational studies carried out have led us to more carefully consider the mechanism of PIP_2_ binding to FAK and the potential effects on FAK activation. When PIP_2_-FAK interactions were first discovered, it was shown that the highly basic KAKTLRK ridge (part of the F2 lobe in the FERM domain) was necessary for PIP_2_-FAK binding *in vitro* and FAK activation *in vivo* [15,27]. More recently, vesicle pull-down and surface plasmon resonance studies identified that the combined kinase and FERM domains of FAK in the autoinhibited conformation bind less effectively to PIP_2_-containing bilayers than the FERM domain alone, indicating that the autoinhibitory conformation of FAK is predisposed to decreasing the favorability of PIP_2_ binding [10]. The motivation for our CGMD studies was to gain molecular insight into these interactions, with the expectation that the interactions would remain localized to the FERM domain. Our simulation faithfully reproduced the known PIP_2_ binding sites located on the basic patch of the FERM domain (Group I). However, observing interactions of Group II residues within the kinase domain with PIP_2_ was completely unexpected. Although the previously-mentioned studies indicate the majority of PIP_2_ binding to FAK occurs within the vicinity of the KAKTLRK ridge, residual binding observed in the KAKTLRK mutant [10] may be due to additional interaction sites as proposed here. Our results indicate that the kinase domain (Group II) could represent a viable site for secondary binding of PIP_2_.

Our simulations indicate that FAK-PIP_2_ interaction is mainly nonspecific due to the electrostatic nature of binding, and agrees well with the lack of influence on FAK activation in the presence of neutrally-charged phospholipids such as DOPC [10,15]. A large number of basic residues on the FAK surface established contact with PIP_2_. The residues engaging frequent contact in each simulation remain largely consistent (S5 Table), indicating a converged identification of PIP_2_-binding residues. Due to the constraints of our protein model, there are potential PIP_2_-interacting residues that may not have been identified by our CGMD simulation. For example, R184 and K190 in the FERM domain (the KAKTLRK ridge), which were shown to be key residues in the allosteric relationship between ATP and PIP_2_ binding [29], and R634 on the kinase domain bridge the Group I and Group II sites but are inaccessible to PIP_2_ in the autoinhibited conformation. However, multiple association and dissociation events were observed, which is supported by the fact that the probability distribution of the principal axis of FAK with respect to the membrane normal showed that no prolonged binding pose existed (S1 Fig). These transient association dynamics mainly stem from the fact that the friction originating from fine-grained degrees of freedom present in an all-atom model is missing in the coarse-grained model used in this study [18,30,31]. Instead of association with a defined binding pocket, PIP_2_ molecules maintain “stable” contact with residues located within Group I and Group II, interacting with the basic surfaces and sampling multiple binding complexes. This observed binding phenomena suggests mutations that could be tested experimentally to further characterize PIP_2_ binding to FAK in the Group II basic patch, in particular R508, R514, K515, K621, and K627.

Our study has provided novel insight into potential PIP_2_ interactions that have been shown to promote the conformational change responsible for FAK activation. The autoinhibitory interaction between the F2 subdomain of the FERM domain and the C-lobe of the kinase domain is formed by a network of charge complementarity, including interactions mediated by the KAKTLRK ridge and R634, a possible secondary PIP_2_-binding target that becomes exposed during separation of the two domains. PIP_2_ binding could potentially weaken the interactions that stabilize the KAKTLRK ridge and R634 in the inactive conformation, leading to conformational change and phosphorylation of Y397. This secondary PIP_2_ binding site may also stabilize FAK in its active conformation. The discovery of a second PIP_2_ binding site on the FAK kinase domain (i.e., Group II) suggests additional mechanisms contributing to conformational change that could operate independently or in concert with the FERM domain. While the initial interaction might resemble the pose illustrated in **Fig 5B**, twisting the domains to simultaneously maximize KAKTLRK ridge and Group II interactions with PIP_2_ could promote conformational change that affects the linker, exposing Y397 for phosphorylation. Further modeling and experimental studies are currently being conducted to fully elucidate the mechanism(s) of FAK activation in response to PIP_2_ binding.

## Supporting Information

S1 Table Percentage of time individual residues interact with PIP_2_ in simulation I using different cutoff values. As the contact cutoff increases, the percentage of time when individual residues make contacts increase accordingly.
(DOCX)

S2 Table Percentage of time individual residues interact with PIP_2_ in simulation II using different cutoff values. (DOCX)

S3 Table Percentage of time individual residues interact with PIP_2_ in simulation III using different cutoff values. (DOCX)

S4 Table Effect of cutoff on the ranking of residues that contact PIP_2._
The residues identified to bind PIP_2_ remain unchanged, meaning our results are insensitive to the cutoff distance.
(DOCX)

S5 Table Comparison of FAK residues that contact PIP_2_ using reported results (Simulations I, II, and III) versus simulation with an initial configuration with FAK bound to bilayer surface. Identified residues in simulation are well-converged. Ignoring the equilibration period in original simulations or starting an independent simulation from a PIP_2_-bound conformation has minimal effects on the amino acid residues from FAK that interact with PIP_2_.
(DOCX)

S1 Fig No preferential binding pose exists between FAK and PIP_2_. Probability (*p*) of the first principal axis of FAK (*V*_*z*_) with respect to the bilayer normal (*z*). The broad distribution indicates there is no preferential binding pose. *black*: simulation I; *red*: simulation II; *green*: simulation III.
(TIFF)

S2 Fig Multiple dissociation and association events occur during FAK-PIP_2_ binding. Interaction energies between FAK and PIP_2_ in simulation I. *red*: van der Waals energy; *black*: electrostatic energy. Several PIP_2_-FAK dissociation and association events occur, as indicated by the significant changes in the interaction energy.
(TIFF)

S3 Fig Group II residues are highly conserved within a representative sample of the animal kingdom. A sequence similarity search was carried out on the human version of focal adhesion kinase 1 (Uniprot Q05397) using the BLAST server [32]. Clustal Omega [33] was used to perform a multiple sequence alignment (MSA) on 25 different species from the animal kingdom (ranging from mammals to sea urchins), with all alignment variables set to their default values. The alignment was visually inspected and manually adjusted in Seaview [34], with the final alignment input into Scorecons [35]. Scorecons was used to quantify residue conservation with respect to the human version of FAK used in the sequence alignment. Each residue in Group II was highly conserved, with the majority being completely conserved. What is interesting to note is that K578 and K621 are both located in flexible loop regions (on the A-loop and near the C-lobe, respectively). When K578 is mutated along with K581 to glutamic acid, FAK’s enzymatic activity is greatly enhanced (‘SuperFAK’ [36]), underscoring the key role K578 plays in activation of FAK, since it is next to the tyrosine residues that are phosphorylated by Src kinase (Y576 and Y577). In addition, the A-loop of FAK kinase was shown to be intimately involved in the allosteric pathway that exists between ATP and PIP_2_ binding recently revealed by all-atom molecular dynamics simulations [37]. The role of K621 in FAK function, if any, is unclear. This residue was in contact with PIP_2_ molecules for the longest duration in our simulations, and this behavior could be linked to the flexible nature of the long loop that connects two of the α-helices in the C-lobe of the kinase domain. Each of the Group II residues (except for K578, which is a well-characterized mutation) appear to be promising candidates for further experimental investigations into the effect they have on binding to PIP_2_. MSA was drawn using the ESPript 3.0 server [38] using default values for residue similarity scores (0.7). Similar residues are shown in bold type with white background, completely conserved residues are shown in white font with black background. *First row*: annotated secondary structure based on PDB 2J0J from avian *Gallus gallus*. *All other rows*: primary amino acid sequences of known FAK1 genes, listed by Uniprot identification numbers. *Green background and black triangles*: Group II residues. Scorecons results for Group II residues are listed below the MSA.
(PDF)
