# Supplementary material for: Novel Phosphotidylinositol 4,5-Bisphosphate Binding Sites on Focal Adhesion Kinase
Source: PLoS One. 2015 Jul 17;10(7):e0132833. doi: 10.1371/journal.pone.0132833 (PMC4505859; doi:10.1371/journal.pone.0132833)
Supplement: S1 Table — As the contact cutoff increases, the percentage of time when individual residues make contacts increase accordingly. (DOCX) [file pone.0132833.s001.docx]

**Table S1.** **Percentage of time individual residues interact with PIP_2_ in simulation I using different cutoff values.**

| cutoff (nm)^a^ | K191 | K216 | K218 | R221 | K222 | R229 | R508 | R514 | K515 | K578 | K621 | K627 | R640 | K657 | R665 |
| --- | --- | --- | --- | --- | --- | --- | --- | --- | --- | --- | --- | --- | --- | --- | --- |
| 0.49 | 6.5 | 7.6 | 16.6 | 0.8 | 16.7 | 14.1 | 6.8 | 3.5 | 15.3 | 23.9 | 18.7 | 29.4 | 15.5 | 4.7 | 8.9 |
| 0.52 | 7.8 | 8.6 | 18.2 | 1.3 | 20.6 | 17.4 | 7.3 | 4.8 | 19.0 | 27.7 | 22.3 | 35.6 | 18.9 | 5.4 | 10.4 |
| 0.55 | 8.2 | 8.9 | 18.7 | 1.5 | 21.5 | 18.2 | 7.5 | 5.0 | 20.0 | 28.8 | 23.3 | 37.1 | 20.0 | 5.6 | 10.9 |
| 0.60 | 8.3 | 8.9 | 18.7 | 1.5 | 21.7 | 18.4 | 7.5 | 5.0 | 20.5 | 29.2 | 23.7 | 37.9 | 20.4 | 5.7 | 11.1 |

^a^ The van der Waals radii between the phosphate bead of PIP_2_ and positively charged side chain bead from ARG/LYS is 0.47 nm.
