# Supplementary material for: Novel Phosphotidylinositol 4,5-Bisphosphate Binding Sites on Focal Adhesion Kinase
Source: PLoS One. 2015 Jul 17;10(7):e0132833. doi: 10.1371/journal.pone.0132833 (PMC4505859; doi:10.1371/journal.pone.0132833)
Supplement: S2 Table — (DOCX) [file pone.0132833.s002.docx]

**Table S2.** **Percentage of time individual residues interact with PIP_2_ in simulation II using different cutoff values.**

| cutoff (nm) | K191 | K216 | K218 | R221 | K222 | R229 | R508 | R514 | K515 | K578 | K621 | K627 | R640 | K657 | R665 |
| --- | --- | --- | --- | --- | --- | --- | --- | --- | --- | --- | --- | --- | --- | --- | --- |
| 0.49 | 3.3 | 4.5 | 9.7 | 1.5 | 5.7 | 3.7 | 24.1 | 8.6 | 10.4 | 30.7 | 42.9 | 31.6 | 13.1 | 5.6 | 9.4 |
| 0.52 | 4.1 | 5.9 | 12.2 | 1.9 | 7.3 | 4.8 | 27.8 | 11.2 | 13.1 | 37.8 | 53.1 | 39.4 | 16.5 | 6.9 | 11.0 |
| 0.55 | 4.4 | 6.4 | 13.0 | 2.0 | 7.9 | 5.1 | 28.6 | 12.0 | 13.8 | 39.4 | 55.5 | 41.7 | 17.7 | 7.3 | 11.5 |
| 0.60 | 4.5 | 6.6 | 13.2 | 2.1 | 8.0 | 5.3 | 28.9 | 12.2 | 14.1 | 40.2 | 56.1 | 42.4 | 18.3 | 7.5 | 11.6 |
