# Supplementary material for: Novel Phosphotidylinositol 4,5-Bisphosphate Binding Sites on Focal Adhesion Kinase
Source: PLoS One. 2015 Jul 17;10(7):e0132833. doi: 10.1371/journal.pone.0132833 (PMC4505859; doi:10.1371/journal.pone.0132833)
Supplement: S3 Table — (DOCX) [file pone.0132833.s003.docx]

**Table S3.** **Percentage of time individual residues interact with PIP_2_ in simulation III using different cutoff values.**

| cutoff (nm) | K191 | K216 | K218 | R221 | K222 | R229 | R508 | R514 | K515 | K578 | K621 | K627 | R640 | K657 | R665 |
| --- | --- | --- | --- | --- | --- | --- | --- | --- | --- | --- | --- | --- | --- | --- | --- |
| 0.49 | 8.5 | 6.4 | 19.0 | 9.4 | 18.4 | 13.3 | 0.0 | 2.8 | 18.6 | 29.5 | 12.9 | 39.6 | 15.7 | 5.6 | 8.5 |
| 0.52 | 11.0 | 7.3 | 23.4 | 10.5 | 21.7 | 16.6 | 0.0 | 3.5 | 22.6 | 35.4 | 15.7 | 48.0 | 19.5 | 6.4 | 10.5 |
| 0.55 | 11.6 | 7.9 | 24.7 | 10.6 | 22.7 | 17.8 | 0.0 | 3.7 | 24.0 | 36.8 | 16.4 | 50.3 | 20.6 | 6.8 | 11.3 |
| 0.60 | 11.9 | 8.0 | 25.0 | 10.6 | 23.1 | 18.2 | 0.0 | 3.7 | 24.6 | 37.3 | 17.0 | 51.1 | 21.2 | 7.0 | 11.7 |
