# Supplementary material for: Novel Phosphotidylinositol 4,5-Bisphosphate Binding Sites on Focal Adhesion Kinase
Source: PLoS One. 2015 Jul 17;10(7):e0132833. doi: 10.1371/journal.pone.0132833 (PMC4505859; doi:10.1371/journal.pone.0132833)
Supplement: S4 Table — The residues identified to bind PIP2 remain unchanged, meaning our results are insensitive to the cutoff distance. (DOCX) [file pone.0132833.s004.docx]

**Table S4.** **Effect of cutoff on the ranking of residues that contact PIP_2._**

|  | Simulation I^a^ | | | | Simulation II | | | | Simulation III | | | |
| --- | --- | --- | --- | --- | --- | --- | --- | --- | --- | --- | --- | --- |
|  | 0.49 | 0.52 | 0.55 | 0.60 | 0.49 | 0.52 | 0.55 | 0.60 | 0.49 | 0.52 | 0.55 | 0.60 |
| 1 | K627 | K627 | K627 | K627 | K621 | K621 | K621 | K621 | K627 | K627 | K627 | K627 |
| 2 | K578 | K578 | K578 | K578 | K627 | K627 | K627 | K627 | K578 | K578 | K578 | K578 |
| 3 | K621 | K621 | K621 | K621 | K578 | K578 | K578 | K578 | K218 | K218 | K218 | K218 |
| 4 | K222 | K222 | K222 | K222 | R508 | R508 | R508 | R508 | K515 | K515 | K515 | K515 |
| 5 | K218 | K515 | K515 | K515 | R640 | R640 | R640 | R640 | K222 | K222 | K222 | K222 |
| 6 | R640 | R640 | R640 | R640 | K515 | K515 | K515 | K515 | R640 | R640 | R640 | R640 |
| 7 | K515 | K218 | K218 | K218 | K218 | K218 | K218 | K218 | R229 | R229 | R229 | R229 |
| 8 | R229 | R229 | R229 | R229 | R665 | R514 | R514 | R426 | K621 | K621 | K621 | K621 |
| 9 | R665 | R665 | R665 | R665 | R550 | R550 | R550 | R550 | R221 | K191 | K191 | K191 |
| 10 | K216 | K216 | K216 | K216 | R514 | R665 | R426 | R514 | R665 | R665 | R665 | R665 |
| 11 | R508 | K191 | K191 | K191 | R426 | R426 | R665 | R665 | K191 | R221 | R221 | R221 |
| 12 | K191 | R508 | R508 | R508 | K222 | K222 | K222 | K222 | K216 | K216 | K216 | K216 |
| 13 | K657 | K657 | K657 | K657 | K587 | K657 | K657 | K657 | K657 | K657 | K657 | K657 |

^a^ Percentage of contact of the listed residue is larger than 5%.
