# Supplementary material for: Novel Phosphotidylinositol 4,5-Bisphosphate Binding Sites on Focal Adhesion Kinase
Source: PLoS One. 2015 Jul 17;10(7):e0132833. doi: 10.1371/journal.pone.0132833 (PMC4505859; doi:10.1371/journal.pone.0132833)
Supplement: S5 Table — Identified residues in simulation are well-converged. Ignoring the equilibration period in original simulations or starting an independent simulation from a PIP2-bound conformation has minimal effects on the amino acid residues from FAK that interact with PIP2. (DOCX) [file pone.0132833.s005.docx]

**Table S5.** **Comparison of FAK residues that contact PIP_2_ using reported results (Simulations I, II, and III) versus simulation with an initial configuration with FAK bound to bilayer surface.**

| Ranking | Simulation I | | Simulation III | | Simulation III | | Bound* |
| --- | --- | --- | --- | --- | --- | --- | --- |
|  | 1 µs | 800 ns^a^ | 1 µs | 800 ns | 1 µs | 800 ns | 1 µs |
| 1^b^ | K627^c^ | K627 | K621 | K621 | K627 | K627 | K218 |
| 2 | K578 | K218 | K627 | K627 | K578 | K578 | K216 |
| 3 | K621 | K578 | K578 | K578 | K218 | K515 | K222 |
| 4 | K222 | K222 | R508 | R508 | K515 | K218 | K191 |
| 5 | K515 | K621 | R640 | R640 | K222 | K222 | R665 |
| 6 | R640 | K515 | K515 | R550 | R640 | R640 | R221 |
| 7 | K218 | R640 | K218 | R514 | R229 | K621 | K657 |
| 8 | R229 | R229 | R514 | R426 | K621 | R229 | K627 |
| 9 | R665 | K216 | R550 | K218 | K191 | K191 | K578 |
| 10 | K216 | R665 | R665 | K515 | R665 | R221 | K621 |
| 11 | K191 | K191 | R426 | R665 | R221 | R665 | R640 |
| 12 | R508 | R508 | K222 | K587 | K216 | K216 | R229 |
| 13 | K657 | R550 | K657 | K216 | K657 | K657 | K587 |

^a^ Analysis carried out on last 800 ns of 1 μs trajectory.

^b^ Among all simulations, despite a shuffle in position, the identified residues for the most part remain the same.

^c^ Listed residues have a % contact time larger than 5%.

*Results from a 1-µs trajectory starting from PIP_2_-bound conformation.
