# Supplementary figures and images for: Novel Phosphotidylinositol 4,5-Bisphosphate Binding Sites on Focal Adhesion Kinase
Source: PLoS One. 2015 Jul 17;10(7):e0132833. doi: 10.1371/journal.pone.0132833 (PMC4505859; doi:10.1371/journal.pone.0132833)

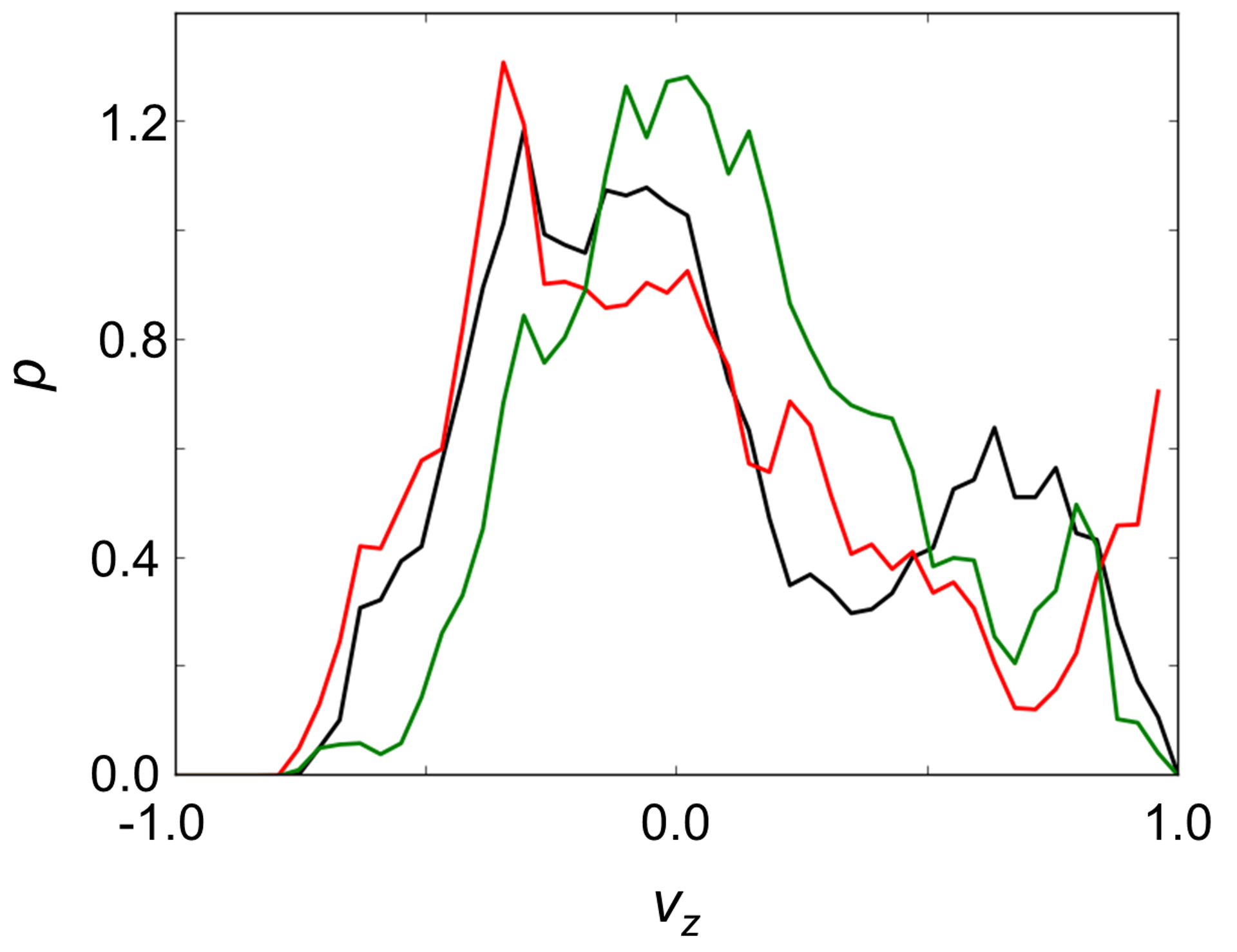

Supplement: S1 Fig — Probability (p) of the first principal axis of FAK (V z) with respect to the bilayer normal (z). The broad distribution indicates there is no preferential binding pose. black: simulation I; red: simulation II; green: simulation III. (TIFF) [file pone.0132833.s006.tiff]

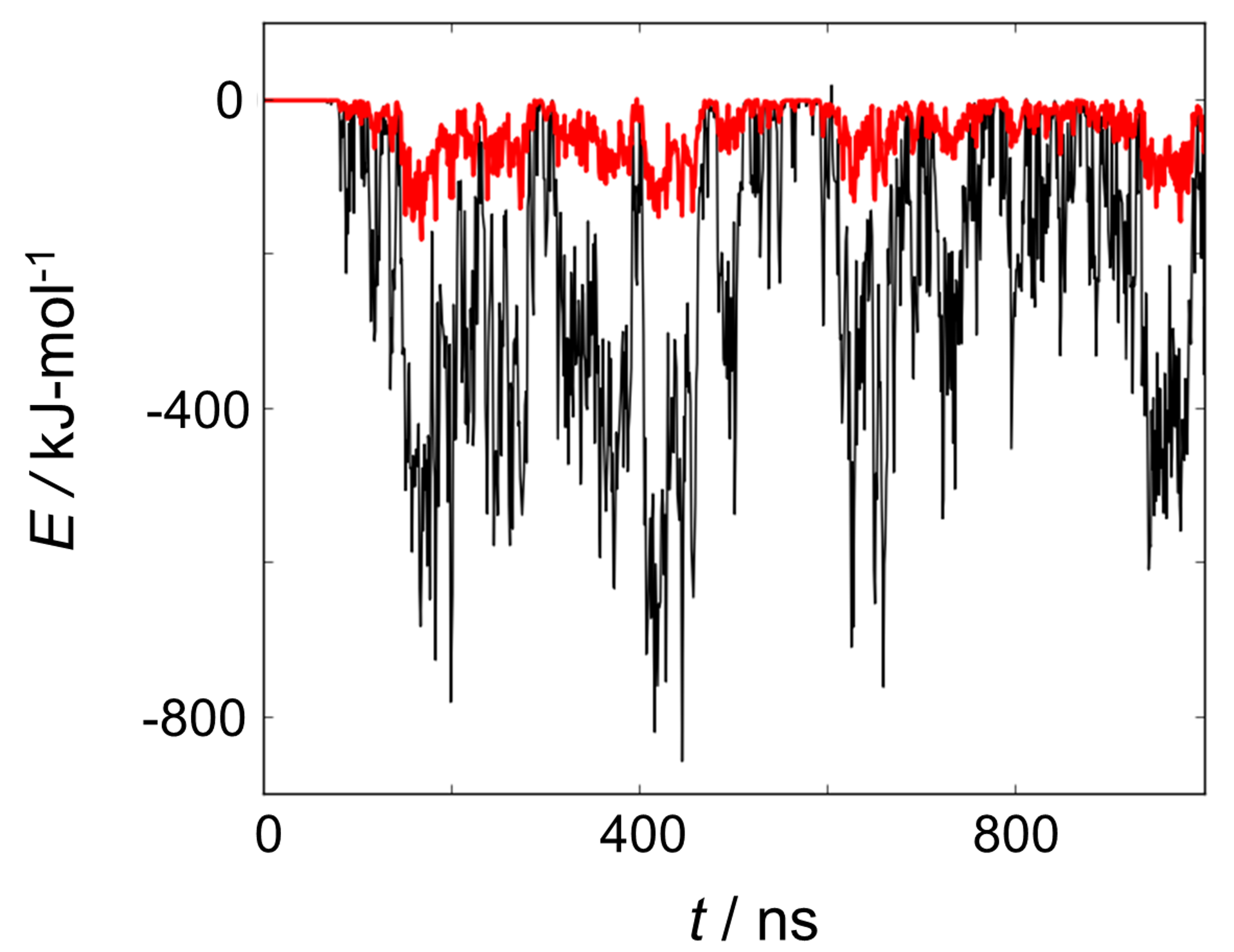

Supplement: S2 Fig — Interaction energies between FAK and PIP2 in simulation I. red: van der Waals energy; black: electrostatic energy. Several PIP2-FAK dissociation and association events occur, as indicated by the significant changes in the interaction energy. (TIFF) [file pone.0132833.s007.tiff]
