# Supplementary material for: Novel Phosphotidylinositol 4,5-Bisphosphate Binding Sites on Focal Adhesion Kinase
Source: PLoS One. 2015 Jul 17;10(7):e0132833. doi: 10.1371/journal.pone.0132833 (PMC4505859; doi:10.1371/journal.pone.0132833)
Supplement: S3 Fig — A sequence similarity search was carried out on the human version of focal adhesion kinase 1 (Uniprot Q05397) using the BLAST server [32]. Clustal Omega [33] was used to perform a multiple sequence alignment (MSA) on 25 different species from the animal kingdom (ranging from mammals to sea urchins), with all alignment variables set to their default values. The alignment was visually inspected and manually adjusted in Seaview [34], with the final alignment input into Scorecons [35]. Scorecons was used to quantify residue conservation with respect to the human version of FAK used in the sequence alignment. Each residue in Group II was highly conserved, with the majority being completely conserved. What is interesting to note is that K578 and K621 are both located in flexible loop regions (on the A-loop and near the C-lobe, respectively). When K578 is mutated along with K581 to glutamic acid, FAK’s enzymatic activity is greatly enhanced (‘SuperFAK’ [36]), underscoring the key role K578 plays in activation of FAK, since it is next to the tyrosine residues that are phosphorylated by Src kinase (Y576 and Y577). In addition, the A-loop of FAK kinase was shown to be intimately involved in the allosteric pathway that exists between ATP and PIP2 binding recently revealed by all-atom molecular dynamics simulations [37]. The role of K621 in FAK function, if any, is unclear. This residue was in contact with PIP2 molecules for the longest duration in our simulations, and this behavior could be linked to the flexible nature of the long loop that connects two of the α-helices in the C-lobe of the kinase domain. Each of the Group II residues (except for K578, which is a well-characterized mutation) appear to be promising candidates for further experimental investigations into the effect they have on binding to PIP2. MSA was drawn using the ESPript 3.0 server [38] using default values for residue similarity scores (0.7). Similar residues are shown in bold type with white background, [file pone.0132833.s008.pdf]

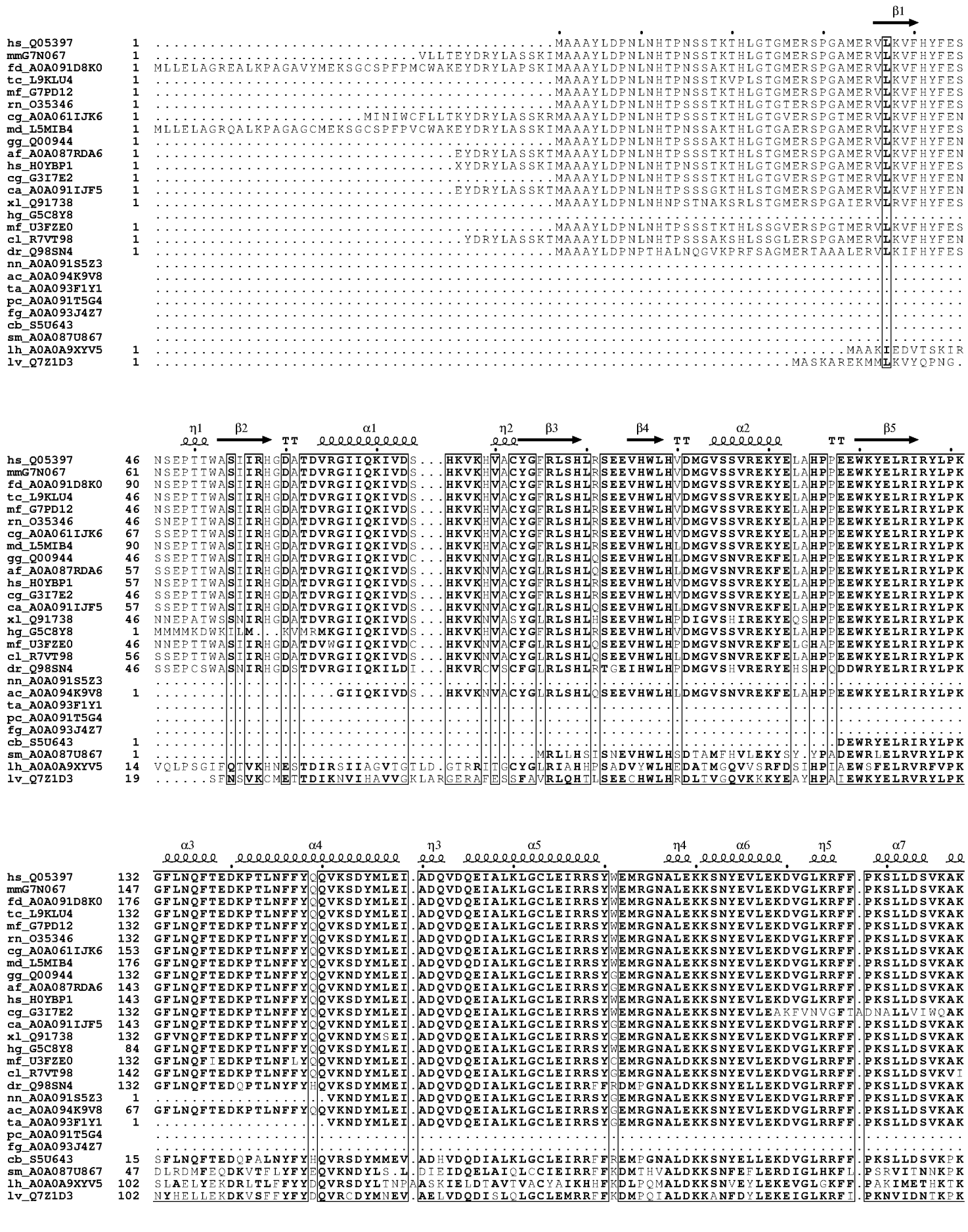
β1
hs_Q05397 1 MAAAYLDPNLNHTPNSSTKTHLGTGMERSPGAMERVLKVFHYFES
mmG7N067 1 VLLTEYDRYLASSKIMAAAYLDPNLNHTPNSSTKTHLGTGMERSPGAMERVLKVFHYFES
fd_A0A091D8K0 1 MLLELAGREALKPAGAVYMEKSGCSPFPMCWAKEYDRYLAPSKIMAAAYLDPNLNHTPNSSAKTHLGTGMERSPGAMERVLKVFHYFES
tc_L9KLU4 1 MAAAYLDPNLNHTPNSSTKVPLSTGMERSPGAMERVLKVFHYFES
mf_G7PD12 1 MAAAYLDPNLNHTPNSSTKTHLGTGMERSPGAMERVLKVFHYFES
rn_O35346 1 MAAAYLDPNLNHTPSSSTKTHLGTGTERSPGAMERVLKVFHYFES
cg_A0A061IJK6 1 MINIWCFLLTKYDRYLASSKRMAAAYLDPNLNHTPSSSTKTHLGTGVERSPGTMERVLKVFHYFEN
md_L5MIB4 1 MLLELAGRQALKPAGAGCMEKSGCSPFPVCWAKEYDRYLASSKIMAAAYLDPNLNHTPNSSAKTHLGTGAERSPGAMERVLKVFHYFES
gg_Q00944 1 MAAAYLDPNLNHTPSSSAKTHLGTGMERSPGAMERVLKVFHYFEN
af_A0A087RDA6 1 EYDRYLASSKTMAAAYLDPNLNHTPSSSTKTHLSTGMERSPGAMERVLKVFHYFEN
hs_H0YBP1 1 XYDRYLASSKIMAAAYLDPNLNHTPNSSTKTHLGTGMERSPGAMERVLKVFHYFES
cg_G3I7E2 1 MAAAYLDPNLNHTPSSSTKTHLGTGVERSPGTMERVLKVFHYFEN
ca_A0A091IJF5 1 EYDRYLASSKTMAAAYLDPNLNHTPSSSGKTHLSTGMERSPGAMERVLKVFHYFEN
xl_Q91738 1 MAAAYLDPNLNHNPSTNAKSRLSTGMERSPGAIERVLRVFHYFES
hg_G5C8Y8
mf_U3FZE0 1 MAAAYLDPNLNHTPSSSTKTHLSSGVERSPGTMERVLKVFHYFES
cl_R7VT98 1 YDRYLASSKTMAAAYLDPNLNHTPSSSAKSHLSSGLERSPGAMERVLKVFHYFEN
dr_Q98SN4 1 MAAAYLDPNPTHALNQGVKPRFSAGMERTAAALERVLKIFHYFES
nn_A0A091S5Z3
ac_A0A094K9V8
ta_A0A093F1Y1
pc_A0A091T5G4
fg_A0A093J4Z7
cb_S5U643
sm_A0A087U867
lh_A0A0A9XYV5 1 MAAKIEDVTSKIR
lv_Q7Z1D3 1 MASKAREKMMLKVYQPNG.
η1 β2 TT α1 η2 β3 β4 TT α2 TT β5
hs_Q05397 46 NSEPTTWASIIRHGDATDVRGIIQKIVDS...HKVKHVACYGFRLSHLRSEEVHWLHVDMGVSSVREKYELAHPPEEWKYELRIRYLPK
mmG7N067 61 NSEPTTWASIIRHGDATDVRGIIQKIVDS...HKVKHVACYGFRLSHLRSEEVHWLHVDMGVSSVREKYELAHPPEEWKYELRIRYLPK
fd_A0A091D8K0 90 NSEPTTWASIIRHGDATDVRGIIQKIVDS...HKVKHVACYGFRLSHLRSEEVHWLHVDMGVSSVREKYELAHPPEEWKYELRIRYLPK
tc_L9KLU4 46 NSEPTTWASIIRHGDATDVRGIIQKIVDS...HKVKHVACYGFRLSHLRSEEVHWLHVDMGVSSVREKYELAHPPEEWKYELRIRYLPK
mf_G7PD12 46 NSEPTTWASIIRHGDATDVRGIIQKIVDS...HKVKHVACYGFRLSHLRSEEVHWLHVDMGVSSVREKYELAHPPEEWKYELRIRYLPK
rn_O35346 46 SNEPTTWASIIRHGDATDVRGIIQKIVDS...HKVKHVACYGFRLSHLRSEEVHWLHVDMGVSSVREKYELAHPPEEWKYELRIRYLPK
cg_A0A061IJK6 67 SSEPTTWASIIRHGDATDVRGIIQKIVDS...HKVKHVACYGFRLSHLRSEEVHWLHLDMGVSSVREKYELAHPPEEWKYELRIRYLPK
md_L5MIB4 90 NSEPTTWASIIRHGDATDVRGIIQKIVDS...HKVKHVACYGFRLSHLRSEEVHWLHLDMGVSSVREKYELAHPPEEWKYELRIRYLPK
gg_Q00944 46 SSEPTTWASIIRHGDATDVRGIIQKIVDC...HKVKNVACYGLRLSHLQSEEVHWLHLDMGVSNVREKFELAHPPEEWKYELRIRYLPK
af_A0A087RDA6 57 SSEPTTWASIIRHGDATDVRGIIQKIVDS...HKVKNVACYGLRLSHLQSEEVHWLHLDMGVSNVREKFELAHPPEEWKYELRIRYLPK
hs_H0YBP1 57 NSEPTTWASIIRHGDATDVRGIIQKIVDS...HKVKHVACYGFRLSHLRSEEVHWLHVDMGVSSVREKYELAHPPEEWKYELRIRYLPK
cg_G3I7E2 46 SSEPTTWASIIRHGDATDVRGIIQKIVDS...HKVKHVACYGFRLSHLRSEEVHWLHVDMGVSSVREKYELAHPPEEWKYELRIRYLPK
ca_A0A091IJF5 57 SSEPTTWASIIRHGDATDVRGIIQKIVDS...HKVKNVACYGLRLSHLQSEEVHWLHLDMGVSNVREKFELAHPPEEWKYELRIRYLPK
xl_Q91738 46 NNEPATWSSNIRHGDATDVRGIIQKIVDS...HKVKNVASYGLRLSHLHSEEVHWLHPDIGVSHIREKYEQSHPPEEWKYELRIRYLPK
hg_G5C8Y8 1 MMMMKDWKILM...KVMRMKGIIQKIVDS...HKVKHVACYGFRLSHLRSEEVHWLHVDMGVSSVREKYELAHPPEEWKYELRIRYLPK
mf_U3FZE0 46 NNEPTTWASIIRHGDATDVWGIIQKIVDC...HKVKNVACFGLRLSHLQSEEVHWLHPDMGVSNVREKFELGHAPEEWKYELRIRYLPK
cl_R7VT98 56 SSEPTTWASIIRHGDATDVRGIIQKIVDS...HKVKNVACYGLRLSHLQSEEVHWLHLDMGVSNVREKFELAHPPEEWKYELRIRYLPK
dr_Q98SN4 46 SSEPCSWASNIRHGDSTDVRGIIQKILDI...HKVRCVSCFGLRLSHLRTGEIHWLHPDMGVSHVRERYEHSHPQDDWRYELRIRYLPK
nn_A0A091S5Z3
ac_A0A094K9V8 1 GIIQKIVDS...HKVKNVACYGLRLSHLQSEEVHWLHLDMGVSNVREKFELAHPPEEWKYELRIRYLPK
ta_A0A093F1Y1
pc_A0A091T5G4
fg_A0A093J4Z7
cb_S5U643 1 DEWRYELRIRYLPK
sm_A0A087U867 1 MRLLHSISNEVHWLHSDTAMFHVLEKYSY.YPADEWRLELRVRYLPK
lh_A0A0A9XYV5 14 VQLPSGIFQTVKHNESTDIRSIIAGVTGTLD.GTRRITGCYGLRIAHHPSADVYWLHEDATMGQVVSRFDSIHPIAEWSFELRVRFVPK
lv_Q7Z1D3 19 ......SFNSVKCMETTDIKNVIHAVVGKLARGERAFESSFAVRLQHTISEECHWLHRDLTVGQVKKKYEAYHPATEWKYELRVRYLPK
α3 α4 η3 α5 η4 α6 η5 α7
hs_Q05397 132 GFLNQFTEDKPTLNFFYQQVKSDYMLEI.ADQVDQEIALKLGCLEIRRSYWEMRGNALEKKSNYEVLEKDVGLKRFF.PKSLLDSVKAK
mmG7N067 147 GFLNQFTEDKPTLNFFYQQVKSDYMLEI.ADQVDQEIALKLGCLEIRRSYWEMRGNALEKKSNYEVLEKDVGLKRFF.PKSLLDSVKAK
fd_A0A091D8K0 176 GFLNQFTEDKPTLNFFYQQVKSDYMLEI.ADQVDQEIALKLGCLEIRRSYWEMRGNALEKKSNYEVLEKDVGLKRFF.PKSLLDSVKAK
tc_L9KLU4 132 GFLNQFTEDKPTLNFFYQQVKSDYMLEI.ADQVDQEIALKLGCLEIRRSYWEMRGNALEKKSNYEVLEKDVGLKRFF.PKSLLDSVKAK
mf_G7PD12 132 GFLNQFTEDKPTLNFFYQQVKSDYMLEI.ADQVDQEIALKLGCLEIRRSYWEMRGNALEKKSNYEVLEKDVGLKRFF.PKSLLDSVKAK
rn_O35346 132 GFLNQFTEDKPTLNFFYQQVKSDYMLEI.ADQVDQDIALKLGCLEIRRSYWEMRGNALEKKSNYEVLEKDVGLKRFF.PKSLLDSVKAK
cg_A0A061IJK6 153 GFLNQFTEDKPTLNFFYQQVKSDYMLEI.ADQVDQEIALKLGCLEIRRSYWEMRGNALEKKSNYEVLEKDVGLKRFF.PKSLLDSVKAK
md_L5MIB4 176 GFLNQFTEDKPTLNFFYQQVKSDYMLEI.ADQVDQDIALKLGCLEIRRSYWEMRGNALEKKSNYEVLEKDVGLKRFF.PRSLLDSVKAK
gg_Q00944 132 GFLNQFTEDKPTLNFFYQQVKNDYMLEI.ADQVDQEIALKLGCLEIRRSYGEMRGNALEKKSNYEVLEKDVGLRRFF.PKSLLDSVKAK
af_A0A087RDA6 143 GFLNQFTEDKPTLNFFYQQVKNDYMLEI.ADQVDQEIALKLGCLEIRRSYGEMRGNALEKKSNYEVLEKDVGLRRFF.PKSLLDSVKAK
hs_H0YBP1 143 GFLNQFTEDKPTLNFFYQQVKSDYMLEI.ADQVDQEIALKLGCLEIRRSYWEMRGNALEKKSNYEVLEKDVGLKRFF.PKSLLDSVKAK
cg_G3I7E2 132 GFLNQFTEDKPTLNFFYQQVKSDYMLEI.ADQVDQEIALKLGCLEIRRSYWEMRGNALEKKSNYEVLEAKFVNVGFTADNALLVIWQAK
ca_A0A091IJF5 143 GFLNQFTEDKPTLNFFYQQVKNDYMLEI.ADQVDQEIALKLGCLEIRRSYGEMRGNALEKKSNYEVLEKDVGLRRFF.PKSLLDSVKAK
xl_Q91738 132 GFVNQFTEDKPTLNFFYQQVKNDYMSEI.ADQVDQEIALKLGCLEIRRSYGEMRGNALEKKSNYEVLEKDVGLKRFF.PKSLLDSVKAK
hg_G5C8Y8 84 GFLNQFTEDKPTLNFFYQQVKSDYMLEI.ADQVDQEIALKLGCLEIRRSYWEMRGNALEKKSNYEVLEKDVGLKRFF.PKSLLDSVKAK
mf_U3FZE0 132 GFLNQFIEDKPTLNFLYQQVKNDYMLEI.ADQVDQEIALKLGCLEIRRSYCEMRGNALDKKSNYEVLEKDVGLRRFF.PKSLLDSVKAK
cl_R7VT98 142 GFLNQFTEDKPTLNFFYQQVKNDYMLEI.ADQVDQEIALKLGCLEIRRSYGEMRGNALEKKSNYEVLEKDVGLRRFF.PKSLLDSVKVI
dr_Q98SN4 132 GFLNQFTEDQPTLNYFYHQVKSDYMMEI.ADQVDQDIALKLGCLEIRRFFRDMPGNALDKKSNYELLEKDVGLRRFF.PKSLLDSVKAK
nn_A0A091S5Z3 1 .VKNDYMLEI.ADQVDQEIALKLGCLEIRRSYGEMRGNALEKKSNYEVLEKDVGLRRFF.PKSLLDSVKAK
ac_A0A094K9V8 67 GFLNQFTEDKPTLNFFYQQVKNDYMLEI.ADQVDQEIALKLGCLEIRRSYGEMRGNALEKKSNYEVLEKDVGLRRFF.PKSLLDSVKAK
ta_A0A093F1Y1 1 .VKNDYMLEI.ADQVDQEIALKLGCLEIRRSYGEMRGNALEKKSNYEVLEKDVGLRRFF.PKSLLDSVKAK
pc_A0A091T5G4
fg_A0A093J4Z7
cb_S5U643 15 SFLNQFTEDQPALNYFYHQVRSDYMMEV.ADHVDQDIALKLGCLEIRRFFREMPGNALDKKSNYELLEKDVGLRRFF.PKSLLDSVKPK
sm_A0A087U867 47 DLRDMFEQDKVTFLYFYFQVKNDYLS.L.DIEIDQELAIQLCCIEIRRFFKDMTHVALDKKSNFEFLERDIGLHKFL.PSRVITNNKPK
lh_A0A0A9XYV5 102 SLAELYEKDRLTLFFYYDQVRSDYLTNPAASKIELDTAVTVACYAIKHHFKDLPQMALDKKSNVEYLEKEVGLGKFF.PAKIMETHKTK
lv_Q7Z1D3 102 NYHELLEKDKVSFFYFYDQVRCDYMNEV.AELVDQDISLQLGCLEMRRFFKDMPQIALDKKANFDYLEKEIGLKRFI.PKNVIDNTKPK

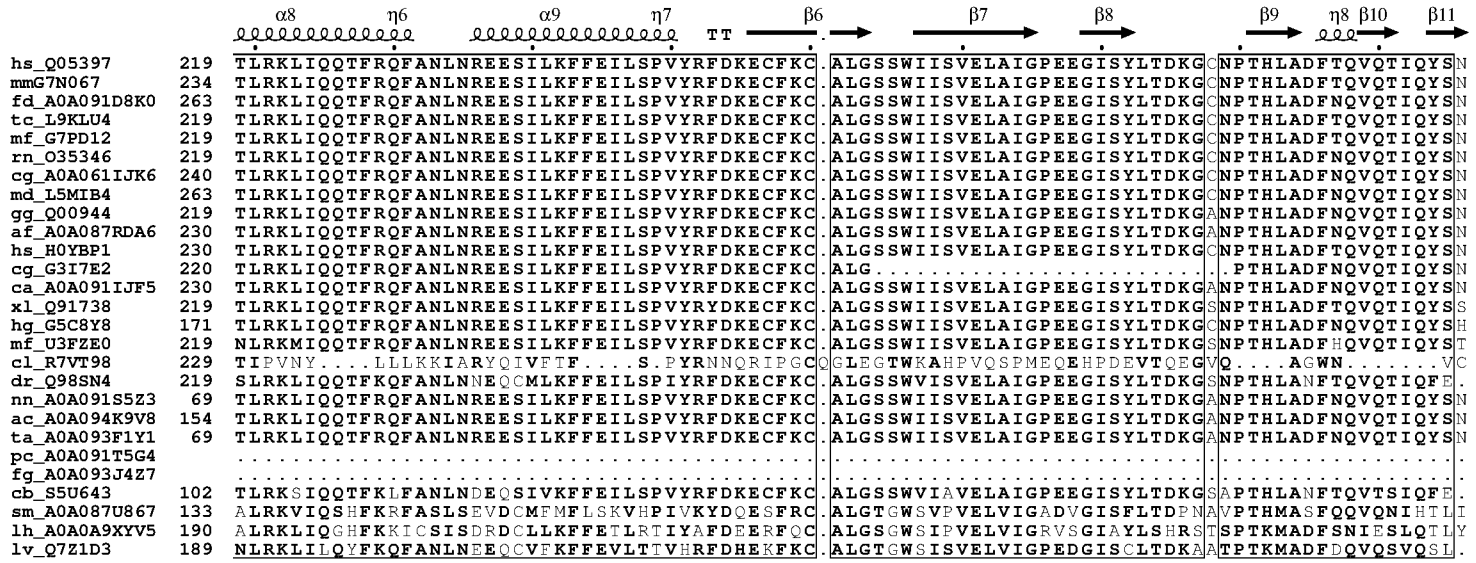
α8 η6 α9 η7 TT β6 β7 β8 β9 η8 β10 β11
hs_Q05397 219 TLRKLIQQTFRQFANLNREESILKFFEILSPVYRFDKECFKC.ALGSSWIISVELAIGPEEGISYLTDKGCNPTHLADFTQVQTIQYSN
mmG7N067 234 TLRKLIQQTFRQFANLNREESILKFFEILSPVYRFDKECFKC.ALGSSWIISVELAIGPEEGISYLTDKGCNPTHLADFTQVQTIQYSN
fd_A0A091D8K0 263 TLRKLIQQTFRQFANLNREESILKFFEILSPVYRFDKECFKC.ALGSSWIISVELAIGPEEGISYLTDKGCNPTHLADFNQVQTIQYSN
tc_L9KLU4 219 TLRKLIQQTFRQFANLNREESILKFFEILSPVYRFDKECFKC.ALGSSWIISVELAIGPEEGISYLTDKGCNPTHLADFTQVQTIQYSN
mf_G7PD12 219 TLRKLIQQTFRQFANLNREESILKFFEILSPVYRFDKECFKC.ALGSSWIISVELAIGPEEGISYLTDKGCNPTHLADFTQVQTIQYSN
rn_O35346 219 TLRKLIQQTFRQFANLNREESILKFFEILSPVYRFDKECFKC.ALGSSWIISVELAIGPEEGISYLTDKGCNPTHLADFNQVQTIQYSN
cg_A0A061IJK6 240 TLRKLIQQTFRQFANLNREESILKFFEILSPVYRFDKECFKC.ALGSSWIISVELAIGPEEGISYLTDKGCNPTHLADFNQVQTIQYSN
md_L5MIB4 263 TLRKLIQQTFRQFANLNREESILKFFEILSPVYRFDKECFKC.ALGSSWIISVELAIGPEEGISYLTDKGCNPTHLADFNQVQTIQYSN
gg_Q00944 219 TLRKLIQQTFRQFANLNREESILKFFEILSPVYRFDKECFKC.ALGSSWIISVELAIGPEEGISYLTDKGANPTHLADFNQVQTIQYSN
af_A0A087RDA6 230 TLRKLIQQTFRQFANLNREESILKFFEILSPVYRFDKECFKC.ALGSSWIISVELAIGPEEGISYLTDKGANPTHLADFNQVQTIQYSN
hs_H0YBP1 230 TLRKLIQQTFRQFANLNREESILKFFEILSPVYRFDKECFKC.ALGSSWIISVELAIGPEEGISYLTDKGCNPTHLADFTQVQTIQYSN
cg_G3I7E2 220 TLRKLIQQTFRQFANLNREESILKFFEILSPVYRFDKECFKC.ALG.....PTHLADFNQVQTIQYSN
ca_A0A091IJF5 230 TLRKLIQQTFRQFANLNREESILKFFEILSPVYRFDKECFKC.ALGSSWIISVELAIGPEEGISYLTDKGANPTHLADFNQVQTIQYSN
xl_Q91738 219 TLRKLIQQTFRQFANLNREESILKFFEILSPVYRYDKECFKC.ALGSSWIISVELAIGPEEGISYLTDKGSNPTHLADFTQVQTIQYSS
hg_G5C8Y8 171 TLRKLIQQTFRQFANLNREESILKFFEILSPVYRFDKECFKC.ALGSSWIISVELAIGPEEGISYLTDKGCNPTHLADFNQVQTIQYSH
mf_U3FZE0 219 NLRKMIQQTFRQFANLNREESILKFFEILSPVYRFDKECFKC.ALGSSWIISVELAIGPEEGISYLTDKGSNPTHLADFHQVQTIQYST
cl_R7VT98 229 TIPVNY....LLLKKIARYQIVFTF....S.PYRNNQRIPGCQGLEGTWKAHPVQSPMEQEHPDEVTQEGVQ....AGWN.....VC
dr_Q98SN4 219 SLRKLIQQTFKQFANLNNEQCMLKFFEILSPIYRFDKECFKC.ALGSSWVISVELAIGPEEGISYLTDKGSNPTHLANFTQVQTIQFE.
nn_A0A091S5Z3 69 TLRKLIQQTFRQFANLNREESILKFFEILSPVYRFDKECFKC.ALGSSWIISVELAIGPEEGISYLTDKGANPTHLADFNQVQTIQYSN
ac_A0A094K9V8 154 TLRKLIQQTFRQFANLNREESILKFFEILSPVYRFDKECFKC.ALGSSWIISVELAIGPEEGISYLTDKGANPTHLADFNQVQTIQYSN
ta_A0A093F1Y1 69 TLRKLIQQTFRQFANLNREESILKFFEILSPVYRFDKECFKC.ALGSSWIISVELAIGPEEGISYLTDKGANPTHLADFNQVQTIQYSN
pc_A0A091T5G4 .....
fg_A0A093J4Z7 .....
cb_S5U643 102 TLRKSIQQTFKLFANLNDEQSIVKFFEILSPVYRFDKECFKC.ALGSSWVIAVELAIGPEEGISYLTDKGSAPTHLANFTQVTSIQFE.
sm_A0A087U867 133 ALRKVIQSHFKRFASLSEVDCMFMFLSKVHPIVKYDQESFRC.ALGTGWSVPVELVIGADVGISFLTDPNAVPTHMASFQQVQNIHTLI
lh_A0A0A9XYV5 190 ALRKLIQGHFKKICSISDRDCLLKFFETLRTIYAFDEERFQC.ALGSGWSIPVELVIGRVSGIAYLSHRSTSPTKMADFSNIESLQTLY
lv_Q7Z1D3 189 NLRKLILQYFKQFANLNEEQCVFKFFEVLTTVHRFDHEKFKC.ALGTGWSISVELVIGPEDGISCLTDKAATPTKMADFDQVQSVQSL.
β12 β13 α10
hs_Q05397 307 SEDK..DRKGMLQLKIAGAPE.....PLTVTAPSLTIAENMADLIDGYCRLVNGTSQSFIIRPQKE.....
mmG7N067 322 SEDK..DRKGMLQLKIAGAPE.....PLTVTAPSLTIAENMADLIDGYCRLVNGASQSFIIRPQKE.....
fd_A0A091D8K0 351 SEDK..DRKGMLQLKIAGAPE.....PLTVTAPSLTIAENMADLIDGYCRLVNGATQSFIIRPQKE.....
tc_L9KLU4 307 SEDK..DRKGMLQLRIAGAPE.....PLTVTAPSLTIAENMADLIDGYCRLVNGATQSFIIRPQKE.....
mf_G7PD12 307 SEDK..DRKGMLQLKIAGAPE.....PLTVTAPSLTIAENMADLIDGYCRLVNGASQSFIIRPQKE.....
rn_O35346 307 SEDK..DRKGMLQLKIAGAPE.....PLTVTAPSLTIAENMADLIDGYCRLVNGATQSFIIRPQKE.....
cg_A0A061IJK6 328 SEDK..DRKGMLQLKIAGAPE.....PLTVTAPSLTIAENMADLIDGYCRLVNGATQSFIIRPQKE.....
md_L5MIB4 351 SEDK..DRKGMLQLKIAGAPE.....PLTVTAPSLTIAENMADLIDGYCRLVNGASQSFIIRPQKE.....
gg_Q00944 307 SEDK..DRKGMLQLKIAGAPE.....PLTVTAPSLTIAENMADLIDGYCRLVNGATQSFIIRPQKE.....
af_A0A087RDA6 318 SEDK..DRKGMLQLKIAGAPE.....PLTVTAPSLTIAENMADLIDGYCRLVNGATQSFIIRPQKE.....
hs_H0YBP1 318 SEDK..DRKGMLQLKIAGAPE.....PLTVTAPSLTIAENMADLIDGYCRLVNGTSQSFIIRPQKE.....
cg_G3I7E2 282 SEDK..DRKGMLQLKIAGAPE.....PLTVTAPSLTIAENMADLIDGYCRLVNGATQSFIIRPQKE.....
ca_A0A091IJF5 318 SEDK..DRKGMLQLKIAGAPE.....PLTVTAPSLTIAENMADLIDGYCRLVNGSTQSFIIRPQKE.....
xl_Q91738 307 SEDK..DRKGMLQLKIAGAPE.....PLTVTAPSLTIAENMADLIDGYCRLVSGASESFIIRPQKE.....
hg_G5C8Y8 259 SEDK..DRKGMLQLKIAGAPE.....PLTVTAPSLTIAENMADLIDGYCRLVNGATQSFIIRPQKE.....
mf_U3FZE0 307 NEDK..DRKGMLQMKIAGAPE.....PLTVTAPSLTIAENMADLIDGYCRLVTGATQSFIIRPQKE.....
cl_R7VT98 298 TEGD..STTSLGSLGQALPPS.....PGTMTAPSLTIAENMADLIDGYCRLVNGATQSFIIRPQKE.....
dr_Q98SN4 306 ..ER..ERKGMLLLDVAGAPE.....PLTVTTTTLNTAENMADLIDGYCRLVSGTSSSFIVRVQKE.....
nn_A0A091S5Z3 157 SEDK..DRKGMLQLKIAGAPE.....PLTVTAPSLTIAENMADLIDGYCRLVNGATQSFIIRPQKE.....
ac_A0A094K9V8 242 SEDK..DRKGMLQLKIAGAPE.....PLTVTAPSLTIAENMADLIDGYCRLVNGATQSFIIRPQKE.....
ta_A0A093F1Y1 157 SEDK..DRKGMLQLKIAGAPE.....PLTVTAPSLTIAENMADLIDGYCRLVNGATQSFIIRPQKE.....
pc_A0A091T5G4 1 .....E.....
fg_A0A093J4Z7 1 .....E.....
cb_S5U643 189 ..EQ..EKKGILQLDVAGAAE.....PLKVTTPCVSTAENMADLIDGYCRLVNKTSLSFIVRVQKE.....
sm_A0A087U867 221 TSDVSSTSKPVLQLKIDGASE.....MLSISCPSASVAESIASLIDGYCQLVHGTKKSFWTQKGGRTSKSRHRNST..
lh_A0A0A9XYV5 278 SGCK.THPKALLRLSVVDASE.....ALTISCANLSEAESLAHLLDGYWRLENPSSDKHIWDRTDSKKS.....
lv_Q7Z1D3 276 STDN..DKKGLLQLKIAGANECDGNFHVYGHAAVDMDSVYITTPSPTIAEDMADLIDGYCRLVLNTEQSLIIRAQQAFNAVTRRSAKKA
β14 η9
hs_Q05397 366 .....GERALPSIPKLANSEKQ.....GMRTHAVSVS.....ETDDYAEIIDEEDTYTMPS.....TRDYE
mmG7N067 381 .....GERALPSIPKLANSEKQ.....GMRTHAVSVS.....ETDDYAEIIDEEDTYTMPS.....TRDYE
fd_A0A091D8K0 410 .....GERALPSIPKLANSEKQ.....GVRTHAVSVS.....ETDDYAEIIDEEDTYTMPS.....TRDYE
tc_L9KLU4 366 .....GERALPSIPKLANSEKQ.....GVRTHAVSVS.....ETDDYAEIIDEEDTYTMPS.....TRDYE
mf_G7PD12 366 .....GERALPSIPKLANSEKQ.....GMRTHAVSVS.....ETDDYAEIIDEEDTYTMPS.....TRDYE
rn_O35346 366 .....GERALPSIPKLANNEKQ.....GMRTHAVSVS.....ETDDYAEIIDEEDTYTMPS.....TRDYE
cg_A0A061IJK6 387 .....GERALPSIPKLANSEKQ.....GIRTHAVSVS.....ETDDYAEIIDEEDTYTMPS.....TRDYE
md_L5MIB4 410 .....GERALPSIPKLANSEKQ.....GVRTHAVSVS.....ETDDYAEIIDEEDTYTMPS.....TRDYE
gg_Q00944 366 .....GERALPSIPKLANNEKQ.....GVRSHTVSVS.....ETDDYAEIIDEEDTYTMPST.....RDYE
af_A0A087RDA6 377 .....GERALPSIPKLANNEKQ.....GVRSHTVSVS.....ETDDYAEIIDEEDTYTMPST.....RDYE
hs_H0YBP1 377 .....GERALPSIPKLANSEKQ.....GMRTHAVSVS.....ETDDYAEIIDEEDTYTMPS.....TRDYE
cg_G3I7E2 341 .....GERALPSIPKLANSEKQ.....GIRTHAVSVS.....ETDDYAEIIDEEDTYTMPS.....TRDYE
ca_A0A091IJF5 377 .....GERALPSIPKLANNEKQ.....GVRSHTVSVS.....ETDDYAEIIDEEDTYTMPST.....RDYE
xl_Q91738 366 .....GERALPSIPKLANNEKH.....GVRPHAVSVSDEFS.....GDETDDYAEIIDEEDTYTMPSKSYGLDEAGDYE
hg_G5C8Y8 318 .....GERALPSIPKLANNEKQ.....GVRTHAVSMSDEIS.....GDETDDYAEIIDEEDTYTMPST.....RDYE
mf_U3FZE0 366 .....GERALPSIPKLANNEKQ.....GIRSHAVSVS.....ETDDYAEIIDEEDTYTMPST.....RDYE
cl_R7VT98 357 .....GERALPSIPKLANNEKQ.....GMRSHTVSVS.....ETDDYAEIIDEEDTYTMPST.....RDYE
dr_Q98SN4 363 .....GERALPSIPKAPKSANHEKRLQGVRAKAISI.....SETDDYAEIIDEEDTYTMPST.....RDYE
nn_A0A091S5Z3 216 .....GERALPSIPKLANNEKQ.....GVRSHTVSVS.....ETDDYAEIIDEEDTYTMPST.....RDYE
ac_A0A094K9V8 301 .....GERALPSIPKLANNEKQ.....GVRSHTVSVS.....ETDDYAEIIDEEDTYTMPST.....RDYE
ta_A0A093F1Y1 216 .....GERALPSIPKLANNEKQ.....GVRSHTVSVS.....ETDDYAEIIDEEDTYTMPST.....RDYE
pc_A0A091T5G4 2 .....GERALPSIPKLANNEKQ.....GVRSHTVSVS.....ETDDYAEIIDEEDTYTMPST.....RDYE
fg_A0A093J4Z7 2 .....GERALPSIPKLANNEKQ.....GVRSHTVSVS.....ETDDYAEIIDEEDTYTMPST.....RDYE
cb_S5U643 246 .....GERALPSLPKAPKASNHDKRLQSVRAKAVSV.....SETDDYAEIIDEEDTYTMPST.....RDYE
sm_A0A087U867 292 .....NFSNLPSSPEK.....KHGFKYGEDYAEIVEEEGDYSTPIV.....KDYE
lh_A0A0A9XYV5 341 .....QEPK.....NSKTKSITSEDYAEIPDEEGDYSTPAT.....KDYE
lv_Q7Z1D3 363 KNAVQDVIQGDRALPSLPGDSRLPSTE....GSNE..VTV....SGRKKIPTVKPSGLSDDYAEIVDDD.DYAMPAA.....KDYE

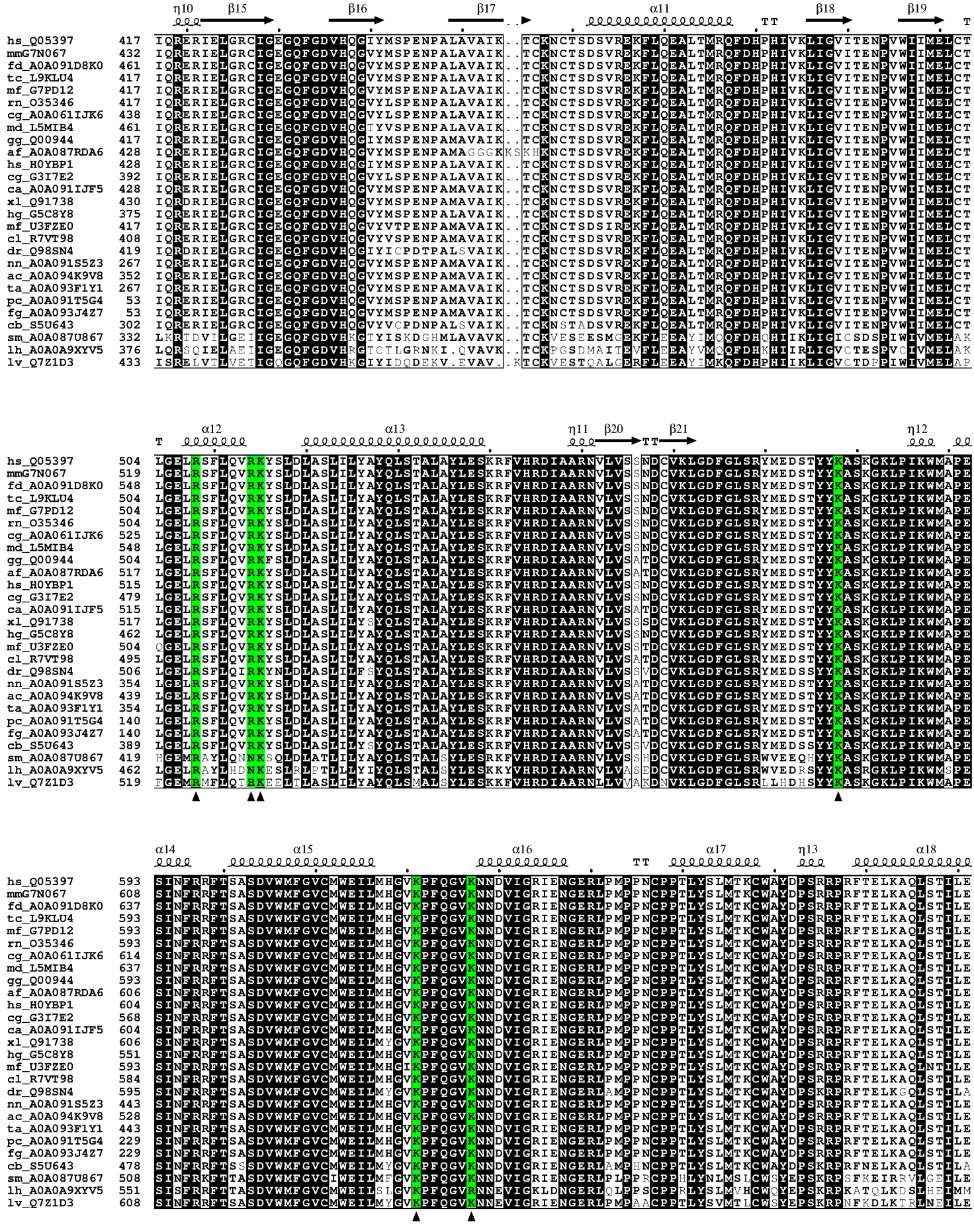
η10 β15 β16 β17 α11 β18 β19
hs_Q05397 417 IQRERIELGRCIGEGQFGDVHQGIYMSPENPALAVAIK..TCKNCTSDSVREKFLQEALTMRQFDHPHIVKLIGVITENPVWIIMELCT
mmG7N067 432 IQRERIELGRCIGEGQFGDVHQGVYMSPENPALAVAIK..TCKNCTSDSVREKFLQEALTMRQFDHPHIVKLIGVITENPVWIIMELCT
fd_A0A091D8K0 461 IQRERIELGRCIGEGQFGDVHQGVYMSPENPALAVAIK..TCKNCTSDSVREKFLQEALTMRQFDHPHIVKLIGVITENPVWIIMELCT
tc_L9KLU4 417 IQRERIELGRCIGEGQFGDVHQGVYMSPENPALAVAIK..TCKNCTSDSVREKFLQEALTMRQFDHPHIVKLIGVITENPVWIIMELCT
mf_G7PD12 417 IQRERIELGRCIGEGQFGDVHQGVYMSPENPALAVAIK..TCKNCTSDSVREKFLQEALTMRQFDHPHIVKLIGVITENPVWIIMELCT
rn_O35346 417 IQRERIELGRCIGEGQFGDVHQGVYLSPENPALAVAIK..TCKNCTSDSVREKFLQEALTMRQFDHPHIVKLIGVITENPVWIIMELCT
cg_A0A061IJK6 438 IQRERIELGRCIGEGQFGDVHQGVYLSPENPALAVAIK..TCKNCTSDSVREKFLQEALTMRQFDHPHIVKLIGVITENPVWIIMELCT
md_L5MIB4 461 IQRERIELGRCIGEGQFGDVHQGTYVSPENPALAVAIK..TCKNCTSDSVREKFLQEALTMRQFDHPHIVKLIGVITENPVWIIMELCT
gg_Q00944 417 IQRERIELGRCIGEGQFGDVHQGIYMSPENPAMAVAIK..TCKNCTSDSVREKFLQEALTMRQFDHPHIVKLIGVITENPVWIIMELCT
af_A0A087RDA6 428 IQRERIELGRCIGEGQFGDVHQGVYMSPENPAMAGGGKKSKHKNCTSDSVREKFLQEALTMRQFDHPHIVKLIGVITENPVWIIMELCT
hs_H0YBP1 428 IQRERIELGRCIGEGQFGDVHQGIYMSPENPALAVAIK..TCKNCTSDSVREKFLQEALTMRQFDHPHIVKLIGVITENPVWIIMELCT
cg_G3I7E2 392 IQRERIELGRCIGEGQFGDVHQGVYLSPENPALAVAIK..TCKNCTSDSVREKFLQEALTMRQFDHPHIVKLIGVITENPVWIIMELCT
ca_A0A091IJF5 428 IQRERIELGRCIGEGQFGDVHQGVYMSPENPAMAVAIK..TCKNCTSDSVREKFLQEALTMRQFDHPHIVKLIGVITENPVWIIMELCT
xl_Q91738 430 IQRDRIELGRCIGEGQFGDVHQGVYMSPENPAMAVAIK..TCKNCTSDSVREKFLQEALTMRQFDHPHIVKLIGVITENPVWIIMELCT
hg_G5C8Y8 375 IQRERIELGRCIGEGQFGDVHQGVYMSPENPALAVAIK..TCKNCTSDSVREKFLQEALTMRQFDHPHIVKLIGVITENPVWIIMELCT
mf_U3FZE0 417 IQRERIELGRCIGEGQFGDVHQGVYVTPENPAMAVAIK..TCKNCTSDSIREKFLQEALTMRQFDHPHIVKLIGVITENPVWIIMELCT
cl_R7VT98 408 IQRERIELGRCIGEGQFGDVHQGVYVSPENPAMAVAIK..TCKNCTSDSVREKFLQEALTMRQFDHPHIVKLIGVITENPVWIIMELCT
dr_Q98SN4 419 IQRDRIELGRCIGEGQFGDVHQGIYICPDTPALSVAIK..TCKNCTSDSVREKFLQEALTMRQFDHPHIVKLIGVITENPVWIIMELCT
nn_A0A091S5Z3 267 IQRERIELGRCIGEGQFGDVHQGVYMSPENPAMAVAIK..TCKNCTSDSVREKFLQEALTMRQFDHPHIVKLIGVITENPVWIIMELCT
ac_A0A094K9V8 352 IQRERIELGRCIGEGQFGDVHQGVYMSPENPAMAVAIK..TCKNCTSDSVREKFLQEALTMRQFDHPHIVKLIGVITENPVWIIMELCT
ta_A0A093F1Y1 267 IQRERIELGRCIGEGQFGDVHQGVYMSPENPAMAVAIK..TCKNCTSDSVREKFLQEALTMRQFDHPHIVKLIGVITENPVWIIMELCT
pc_A0A091T5G4 53 IQRERIELGRCIGEGQFGDVHQGVYMSPENPAMAVAIK..TCKNCTSDSVREKFLQEALTMRQFDHPHIVKLIGVITENPVWIIMELCT
fg_A0A093J4Z7 53 IQRERIELGRCIGEGQFGDVHQGVYMSPENPAMAVAIK..TCKNCTSDSVREKFLQEALTMRQFDHPHIVKLIGVITENPVWIIMELCT
cb_S5U643 302 IQRERIELGRCIGEGQFGDVHQGVYVCPDNPALSVAIK..TCKNSTADSVREKFLQEALTMRQFDHPHIVKLIGVITENPVWIIMELCT
sm_A0A087U867 332 LKRTDVILGETIGEGQFGDVHKGTYISKDGHMLAVAVK..TCKVESEESMGEKFLEEAYIMQQFDHQHIIKLIGICSDSPIWIVMELAK
lh_A0A0A9XYV5 376 LQRSQIELAETIGEGQFGDVHRGTCTLGRNKI.QVAVK..TCKPGSDMAITEVFLEEAYVMQQFDHKHIIRLIGVCTESPVCIVMELAK
lv_Q7Z1D3 433 ISRELVTLVETIGQGQFGDVHKGIYIDQDEKV.EVAV..KTCKVESTQALGERFLEEAYIMKQFDHPHIIKLIGVCTDPPIWIVMELAP
T α12 α13 η11 β20 β21 η12
hs_Q05397 504 LGELRSFLQVRKYSLDLASLILYAYQLSTALAYLESKRFVHRDIAARNVLVSSNDCVKLGDFGLSRYMEDSTYYKASKGKLPIKWMAPE
mmG7N067 519 LGELRSFLQVRKYSLDLASLILYAYQLSTALAYLESKRFVHRDIAARNVLVSSNDCVKLGDFGLSRYMEDSTYYKASKGKLPIKWMAPE
fd_A0A091D8K0 548 LGELRSFLQVRKYSLDLASLILYAYQLSTALAYLESKRFVHRDIAARNVLVSSNDCVKLGDFGLSRYMEDSTYYKASKGKLPIKWMAPE
tc_L9KLU4 504 LGELRSFLQVRKYSLDLASLILYAYQLSTALAYLESKRFVHRDIAARNVLVSSNDCVKLGDFGLSRYMEDSTYYKASKGKLPIKWMAPE
mf_G7PD12 504 LGELRSFLQVRKYSLDLASLILYAYQLSTALAYLESKRFVHRDIAARNVLVSSNDCVKLGDFGLSRYMEDSTYYKASKGKLPIKWMAPE
rn_O35346 504 LGELRSFLQVRKYSLDLASLILYAYQLSTALAYLESKRFVHRDIAARNVLVSSNDCVKLGDFGLSRYMEDSTYYKASKGKLPIKWMAPE
cg_A0A061IJK6 525 LGELRSFLQVRKYSLDLASLILYAYQLSTALAYLESKRFVHRDIAARNVLVSSNDCVKLGDFGLSRYMEDSTYYKASKGKLPIKWMAPE
md_L5MIB4 548 LGELRSFLQVRKYSLDLASLILYAYQLSTALAYLESKRFVHRDIAARNVLVSSNDCVKLGDFGLSRYMEDSTYYKASKGKLPIKWMAPE
gg_Q00944 504 LGELRSFLQVRKFSLDLASLILYAYQLSTALAYLESKRFVHRDIAARNVLVSATDCVKLGDFGLSRYMEDSTYYKASKGKLPIKWMAPE
af_A0A087RDA6 517 LGELRSFLQVRKYSLDLASLILYAYQLSTALAYLESKRFVHRDIAARNVLVSATDCVKLGDFGLSRYMEDSTYYKASKGKLPIKWMAPE
hs_H0YBP1 515 LGELRSFLQVRKYSLDLASLILYAYQLSTALAYLESKRFVHRDIAARNVLVSSNDCVKLGDFGLSRYMEDSTYYKASKGKLPIKWMAPE
cg_G3I7E2 479 LGELRSFLQVRKYSLDLASLILYAYQLSTALAYLESKRFVHRDIAARNVLVSSNDCVKLGDFGLSRYMEDSTYYKASKGKLPIKWMAPE
ca_A0A091IJF5 515 LGELRSFLQVRKYSLDLASLILYAYQLSTALAYLESKRFVHRDIAARNVLVSATDCVKLGDFGLSRYMEDSTYYKASKGKLPIKWMAPE
xl_Q91738 517 LGELRSFLQVRKYSLDLASLILYSYQLSTALAYLESKRFVHRDIAARNVLVSSSDCVKLGDFGLSRYMEDSTYYKASKGKLPIKWMAPE
hg_G5C8Y8 462 LGELRSFLQVRKYSLDLASLILYAYQLSTALAYLESKRFVHRDIAARNVLVSSNDCVKLGDFGLSRYMEDSTYYKASKGKLPIKWMAPE
mf_U3FZE0 504 QGELRSFLQVRKYSLDLASLILYAYQLSTALAYLESKRFVHRDIAARNVLVSSTDCVKLGDFGLSRYMEDSTYYKASKGKLPIKWMAPE
cl_R7VT98 495 LGELRSFLQVRKYSLDLASLILYAYQLSTALAYLESKRFVHRDIAARNVLVSATDCVKLGDFGLSRYMEDSTYYKASKGKLPIKWMAPE
dr_Q98SN4 506 LGELRSFLQIRKYNLDLASLILFSYQLSTALAYLESKRFVHRDIAARNVLVSSVDCVKLGDFGLSRYMEDSSYYKASKGKLPIKWMAPE
nn_A0A091S5Z3 354 LGELRSFLQVRKYSLDLASLILYAYQLSTALAYLESKRFVHRDIAARNVLVSATDCVKLGDFGLSRYMEDSTYYKASKGKLPIKWMAPE
ac_A0A094K9V8 439 LGELRSFLQVRKYSLDLASLILYAYQLSTALAYLESKRFVHRDIAARNVLVSATDCVKLGDFGLSRYMEDSTYYKASKGKLPIKWMAPE
ta_A0A093F1Y1 354 LGELRSFLQVRKYSLDLASLILYAYQLSTALAYLESKRFVHRDIAARNVLVSATDCVKLGDFGLSRYMEDSTYYKASKGKLPIKWMAPE
pc_A0A091T5G4 140 LGELRSFLQVRKYSLDLASLILYAYQLSTALAYLESKRFVHRDIAARNVLVSATDCVKLGDFGLSRYMEDSTYYKASKGKLPIKWMAPE
fg_A0A093J4Z7 140 LGELRSFLQVRKYSLDLASLILYAYQLSTALAYLESKRFVHRDIAARNVLVSATDCVKLGDFGLSRYMEDSTYYKASKGKLPIKWMAPE
cb_S5U643 389 LGELRSFLQVRKYSLDLASLILYSYQLSTALAYLESKRFVHRDIAARNVLVSSVDCVKLGDFGLSRYMEDSSYYKASKGKLPIKWMAPE
sm_A0A087U867 419 HGEMRAYLQNNKSQLDLATLILYAYQLSTALSYLESRKFVHRDIAARNVLVSSHDCVKLGDFGLSRWVEEQHYYKASKGKLPIKWMAPE
lh_A0A0A9XYV5 462 LGELRAYLHDNKESLRLPTLLLYIYQLSTALSYLESKKYVHRDIAARNVLVASEDCVKLGDFGLSRWVEDRSYYKASRGKLPIKWMSPE
lv_Q7Z1D3 519 FGEMRMFLQTHKEELTLASLILYAYQLSMALSYLESKKFVHRDIAARNLLVVAKDNVKLGDFGLSRLLHDHSYYKASKGKLPIKWMAPE
α14 α15 α16 α17 η13 α18
hs_Q05397 593 SINFRRFTSASDVWMFGVCMWEILMHGVKPFQGVKNNDVIGRIENGERLPMPPNCPPTLYSLMTKCWAYDPSRRPRFTELKAQLSTILE
mmG7N067 608 SINFRRFTSASDVWMFGVCMWEILMHGVKPFQGVKNNDVIGRIENGERLPMPPNCPPTLYSLMTKCWAYDPSRRPRFTELKAQLSTILE
fd_A0A091D8K0 637 SINFRRFTSASDVWMFGVCMWEILMHGVKPFQGVKNNDVIGRIENGERLPMPPNCPPTLYSLMTKCWAYDPSRRPRFTELKAQLSTILE
tc_L9KLU4 593 SINFRRFTSASDVWMFGVCMWEILMHGVKPFQGVKNNDVIGRIENGERLPMPPNCPPTLYSLMTKCWAYDPSRRPRFTELKAQLSTILE
mf_G7PD12 593 SINFRRFTSASDVWMFGVCMWEILMHGVKPFQGVKNNDVIGRIENGERLPMPPNCPPTLYSLMTKCWAYDPSRRPRFTELKAQLSTILE
rn_O35346 593 SINFRRFTSASDVWMFGVCMWEILMHGVKPFQGVKNNDVIGRIENGERLPMPPNCPPTLYSLMTKCWAYDPSRRPRFTELKAQLSTILE
cg_A0A061IJK6 614 SINFRRFTSASDVWMFGVCMWEILMHGVKPFQGVKNNDVIGRIENGERLPMPPNCPPTLYSLMTKCWAYDPSRRPRFTELKAQLSTILE
md_L5MIB4 637 SINFRRFTSASDVWMFGVCMWEILMHGVKPFQGVKNNDVIGRIENGERLPMPPNCPPTLYSLMTKCWAYDPSRRPRFTELKAQLSTILE
gg_Q00944 593 SINFRRFTSASDVWMFGVCMWEILMHGVKPFQGVKNNDVIGRIENGERLPMPPNCPPTLYSLMTKCWAYDPSRRPRFTELKAQLSTILE
af_A0A087RDA6 606 SINFRRFTSASDVWMFGVCMWEILMHGVKPFQGVKNNDVIGRIENGERLPMPPNCPPTLYSLMTKCWAYDPSRRPRFTELKAQLSTILE
hs_H0YBP1 604 SINFRRFTSASDVWMFGVCMWEILMHGVKPFQGVKNNDVIGRIENGERLPMPPNCPPTLYSLMTKCWAYDPSRRPRFTELKAQLSTILE
cg_G3I7E2 568 SINFRRFTSASDVWMFGVCMWEILMHGVKPFQGVKNNDVIGRIENGERLPMPPNCPPTLYSLMTKCWAYDPSRRPRFTELKAQLSTILE
ca_A0A091IJF5 604 SINFRRFTSASDVWMFGVCMWEILMHGVKPFQGVKNNDVIGRIENGERLPMPPNCPPTLYSLMTKCWAYDPSRRPRFTELKAQLSTILE
xl_Q91738 606 SINFRRFTSASDVWMFGVCMWEILMYGVKPFQGVKNNDVIGRIENGERLPMPPNCPPTLYSLMTKCWAYDPSRRPRFTELKAQLSTILE
hg_G5C8Y8 551 SINFRRFTSASDVWMFGVCMWEILMHGIKPFQGVKNNDVIGRIENGERLPMPPNCPPTLYSLMTKCWAYDPSRRPRFTELKAQLSTILE
mf_U3FZE0 593 SINFRRFTSASDVWMFGVCMWEILMHGVKPFQGVKNNDVIGRIENGERLPMPPNCPPTLYSLMTKCWAYDPSRRPRFTELKAQLNTILE
cl_R7VT98 584 SINFRRFTSASDVWMFGVCMWEILMHGVKPFQGVKNNDVIGRIENGERLPMPPNCPPTLYSLMTKCWAYDPSRRPRFTELKAQLSTILE
dr_Q98SN4 595 SINFRRFTSASDVWMFGVCMWEILMYGVKPFQGVKNNDVIGRIENGERLAMPPNCPPTLYSLMTKCWAYDPSKRPRFTELKGQLSTILA
nn_A0A091S5Z3 443 SINFRRFTSASDVWMFGVCMWEILMHGVKPFQGVKNNDVIGRIENGERLPMPPNCPPTLYSLMTKCWAYDPSRRPRFTELKAQLSTILE
ac_A0A094K9V8 528 SINFRRFTSASDVWMFGVCMWEILMHGVKPFQGVKNNDVIGRIENGERLPMPPNCPPTLYSLMTKCWAYDPSRRPRFTELKAQLSTILE
ta_A0A093F1Y1 443 SINFRRFTSASDVWMFGVCMWEILMHGVKPFQGVKNNDVIGRIENGERLPMPPNCPPTLYSLMTKCWAYDPSRRPRFTELKAQLSTILE
pc_A0A091T5G4 229 SINFRRFTSASDVWMFGVCMWEILMHGVKPFQGVKNNDVIGRIENGERLPMPPNCPPTLYSLMTKCWAYDPSRRPRFTELKAQLSTILE
fg_A0A093J4Z7 229 SINFRRFTSASDVWMFGVCMWEILMHGVKPFQGVKNNDVIGRIENGERLPMPPNCPPTLYSLMTKCWAYDPSRRPRFTELKAQLSTILE
cb_S5U643 478 SINFRRFTSSSDVWMFGVCMWEILMYGVKPFQGVKNNDVIGRIENGERLAMPHNCPPTLYSLMTKCWAYDPSKRPRFNELKAQLSTILA
sm_A0A087U867 508 SINFRKFTTASDVWMFGVCIWEILMFGVKPFQGVKNNDVIGRIENGERLPLPPRCPPHLYNLMSLCWSYEPSKRPSFKEIRRVLGEILE
lh_A0A0A9XYV5 551 SISFRRFTTASDVWMFGVCMWEILSLGVKPFQGVKNNEVIGKLDNGERLQLPPSCPPRLYSLMVHCWQYEPSKRPKATQLKDSLHEIMM
lv_Q7Z1D3 608 SINFRRFTSASDVWMFGVCMWEILMYGVKPFQGVKNNEVIGRIENGERLPMPAACPPTLYSVMTLCWSYEPSKRPNFKDLKTRLNEILE

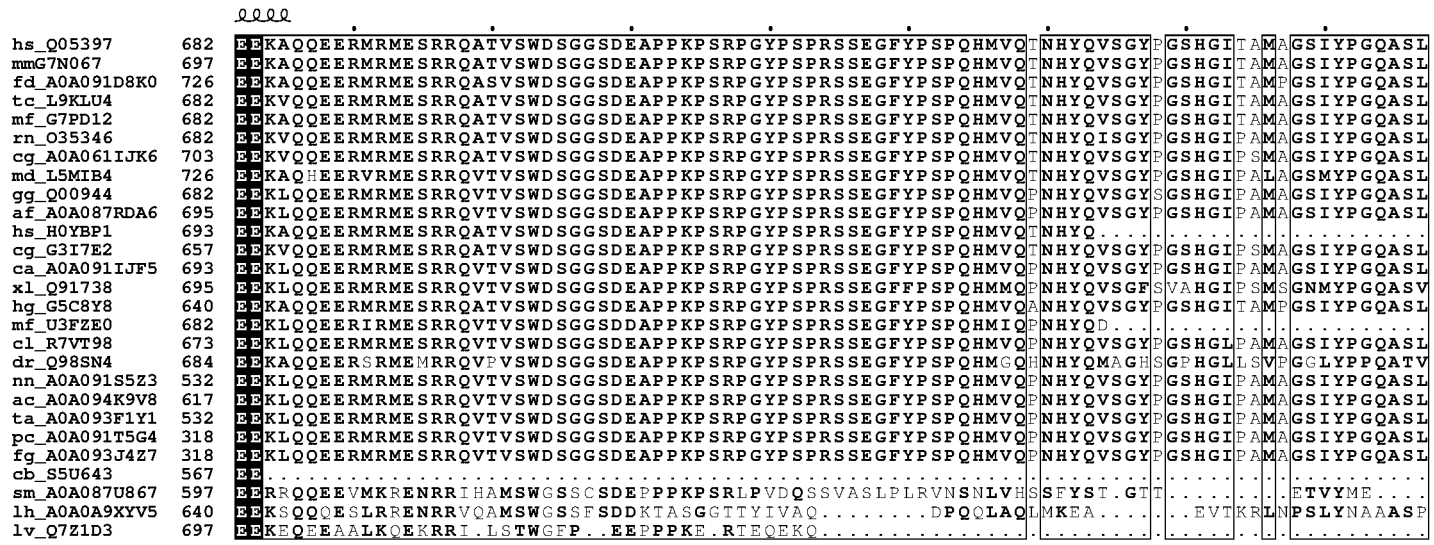


▲= Group II amino acid residues. Residue conservation with respect to human version of FAK (hs_Q05397) according to Scorecons is as follows: R508 = 100%; R514 = 78.5%; K515 = 100%; K578 = 100%; K621 = 100%; K627 = 95%.
